# Circular RNA circ-MTHFD1L induces HR repair to promote gemcitabine resistance via the miR-615-3p/RPN6 axis in pancreatic ductal adenocarcinoma

**DOI:** 10.1186/s13046-022-02343-z

**Published:** 2022-04-23

**Authors:** Zhi-Wen Chen, Jian-Fei Hu, Zu-Wei Wang, Cheng-Yu Liao, Feng-Ping Kang, Cai-Feng Lin, Yi Huang, Long Huang, Yi-Feng Tian, Shi Chen

**Affiliations:** 1 Shengli Clinical Medical College of Fujian Medical University, Fuzhou, 350001 Fujian Province People’s Republic of China; 2 Department of Hepatobiliary Surgery, Jinshan Branch of Fujian Province Hospital, Fuzhou, 350007 Fujian Province People’s Republic of China; 3 Center for Experimental Research in Clinical Medicine, Fujian Provincial Hospital, Fuzhou, 350001 Fujian Province People’s Republic of China; 4 Department of Hepatopancreatobiliary Surgery, Fujian Provincial Hospital, Fujian Medical University, No. 134, East Street, Fuzhou, 350001 Fujian Province People’s Republic of China

**Keywords:** Circ-MTHFD1L, miR-615-3p, RPN6, Gemcitabine resistance, Pancreatic cancer

## Abstract

**Background:**

Chemoresistance of pancreatic cancer is the main reason for the poor treatment effect of pancreatic cancer patients. Exploring chemotherapy resistance-related genes has been a difficult and hot topic of oncology. Numerous studies implicate the key roles of circular RNAs (circRNAs) in the development of pancreatic cancer. However, the regulation of circRNAs in the process of pancreatic ductal adenocarcinoma (PDAC) chemotherapy resistance is not yet fully clear.

**Methods:**

Based on the cross-analysis of the Gene Expression Omnibus (GEO) database and the data of our center, we explored a new molecule, hsa_circ_0078297 (circ-MTHFD1L), related to chemotherapy resistance. QRT-PCR was used to detect the expression of circRNAs, miRNAs, and mRNAs in human PDAC tissues and their matched normal tissues. The interaction between circ-MTHFD1L and miR-615-3p/RPN6 signal axis was confirmed by a series of experiments such as Dual-luciferase reporter assay, fluorescence in situ hybridization (FISH) RNA immunoprecipitation (RIP) assays.

**Results:**

Circ-MTHFD1L was significantly increased in PDAC tissues and cells. And in PDAC patients, the higher the expression level of circ-MTHFD1L, the worse the prognosis. Mechanism analysis showed that circ-MTHFD1L, as an endogenous miR-615-3p sponge, upregulates the expression of RPN6, thereby promoting DNA damage repair and exerting its effect on enhancing gemcitabine chemotherapy resistance. More importantly, we also found that Silencing circ-MTHFD1L combined with olaparib can increase the sensitivity of pancreatic cancer to gemcitabine.

**Conclusion:**

Circ-MTHFD1L maintains PDAC gemcitabine resistance through the miR-615-3p/RPN6 signal axis. Circ-MTHFD1L may be a molecular marker for the effective treatment of PDAC.

**Supplementary Information:**

The online version contains supplementary material available at 10.1186/s13046-022-02343-z.

## Background

Pancreatic cancer is known as the “king of cancer”. It has a high malignancy and a very poor prognosis. The overall morbidity and mortality rates worldwide are increasing year by year. It is estimated that pancreatic cancer will become the second leading cause of death related to malignant tumors in humans by 2030 [1]. The current consensus of the world’s authoritative pancreatic cancer experts shows that the existing treatments for pancreatic cancer have reached a therapeutic plateau, and it is difficult to make a breakthrough in the short term. The 5-year survival rate is only 9%, the lowest among all malignant tumors. The main reason is that pancreatic cancer has the characteristics of low surgical resection rate, high recurrence and metastasis rate, and strong drug resistance [2–4]. Although various new treatment methods have been developed rapidly in recent years, they are still less effective for pancreatic cancer, especially advanced pancreatic cancer [5–8], and chemotherapy drugs are still the cornerstone of pancreatic cancer treatment. It is inevitable for patients with pancreatic cancer to receive chemotherapy, and the status of gemcitabine for pancreatic cancer chemotherapy is unshakable [9, 10]. The focus of pancreatic cancer treatment is chemotherapy, and the difficulty of chemotherapy lies in chemoresistance. Therefore, it is urgent to explore the potential mechanism of PDAC gemcitabine chemotherapy resistance and find new and effective molecular diagnostic markers and therapeutic targets, which are significant for improving the survival rate and quality of life of patients with pancreatic cancer.

Circular RNAs (circRNAs), a rich variety of non-coding RNAs (ncRNAs), are mostly generated from the ‘back-splicing’ of precursor mRNA (pre-mRNA) transcripts [11]. They have a continuous closed structure without 5′ to 3′ polarity or 3′ Polya tail, which has become a hot topic in RNA aspect research [12]. The unique circular structure of circRNAs gives them inherent resistance to exonuclease degradation and is more stable than linear RNA [13]. In recent years, extensive studies have found that circRNAs play a key role in tumorigenesis by acting as competing endogenous RNAs, such as liver cancer [14], lung cancer [15], Gastric cancer [16], breast cancer [17]. Therefore, circRNAs have great potential as a valuable tumor diagnostic biomarker or therapeutic target [18]. circRNAs also play a key role in pancreatic cancer. The latest review report summarizes that circRNAs regulate pancreatic cancer cell processes, including proliferation, apoptosis, cell cycle, EMT, angiogenesis [19, 20]. However, the role of circRNAs in the chemoresistance of pancreatic cancer still has a great vacancy. It is of clinical significance to explore the role and mechanism of the new circRNAs.

In this study, we screened the differentially expressed circRNAs in human pancreatic cancer cell lines and gemcitabine resistant pancreatic cancer cell lines through circRNA sequencing technology, cross-screened with data from the authoritative public database GEO, and discovered a new gemcitabine resistant-related molecule in pancreatic cancer: hsa_circ_0078297 (circ-MTHFD1L). We found that circ-MTHFD1L, as an endogenous miR-615-3p sponge, upregulates the expression of RPN6 and then regulates the expression of BRCA1/2, thus exerting its anti-drug effect. Studies have proved that olaparib has practical clinical value for patients with gBRCA mutation [21]. However, some “Tumors with BRCA-like mutations” have been discovered recently: they do not have BRCA gene mutations, but the molecular mechanism of tumor canceration is similar, so PARP inhibitors are also suitable [22, 23]. We have preliminarily confirmed through in vivo experiments in nude mice that olaparib can enhance the therapeutic effect of gemcitabine on pancreatic cancer after silencing circ-MTHFD1L. It may become a molecular biomarker for BRCAness, the first report on the expression, role, and regulation mechanism of circ-MTHFD1L in tumors. It is suggested that circ-MTHFD1L may be a new potential molecular marker for chemotherapy resistance of pancreatic cancer gemcitabine and a potential therapeutic target for combined treatment with olaparib.

## Materials and methods

### Patient information and tissue specimens

Ninety six pairs of pancreatic cancer tissues and normal tissues from gemcitabine-treated PADC patients, which were histopathologically and clinically diagnosed, were obtained from Fujian Provincial Hospital. All experiments involving human samples and clinical data were approved by the Accreditation Committee of Fujian Provincial Hospital.

### Immunohistochemistry

For Immunohistochemistry staining, slides were incubated with primary antibodies targeting RPN6 (Cat. No. ER64693, HUABIO) overnight at 4 °C, followed by incubation with secondary antibodies at room temperature for 30 min. Next, sections were stained with DAB solution for 10 min. The staining intensities of RPN6 were assigned as follows: 0, negative; 1, weak; 2, medium; and 3, strong. The positive rate of tumor cells was scored as follows: 1, 0–25%; 2, 26–50%; 3, 51–75%; and 4, 76–100%. The IHC scores were calculated by multiplying the staining intensity and positive rate scores. The sections were reviewed by two pathologists.

### Establishment of cell lines and transfection

Human PDAC cells (PATU8988T, AsPC-1, BxPC-3, PANC-1, and SW1990) and human pancreatic ductal epithelial (HPDE) cells were obtained from American Type Culture Collection (ATCC, Manassas, VA, USA). PANC-1 cells were cultured in Dulbecco’s modified Eagle’s medium (DMEM) supplemented with 10% FBS and 2 mM L-glutamine. BxPC-3 cells were maintained in RPMI 1640 medium supplemented with 2.5 g/L glucose, 1 mM sodium pyruvate, and 10% fetal bovine serum. Cells were cultured in a 37 °C, 5% CO_2_ incubator with routine mycoplasma check once every month. Gemcitabine-resistant PANC-1 (PANC-1-GR) cells and gemcitabine-resistant BxPC-3 (BxPC-3-GR) cells were established by our laboratory. Briefly, PANC-1 cells were treated with gemcitabine by a stepwise increase in drug concentration from 1 to 80 μM (1, 5, 10, 20, 40, and 80) every 2 weeks, until cells became resistant to 80 μM of gemcitabine. In contrast, BxPC-3 cells were exposed to gemcitabine ranging from 0.5 to 40 μM (0.5, 2, 5, 10, 20, and 40) every 2 weeks until cells became resistant to 40 μM of gemcitabine. The miR-615-3p mimic, inhibitor, and corresponding control oligonucleotides were purchased from Shangya (Fuzhou, China). The transfection of miR-615-3p mimics, inhibitor, or corresponding NCs into cells was performed using Lipofectamine 3000 (Invitrogen). Lentivirus shRNAs were constructed by Genechem (Shanghai, China). The cells were transfected with lentivirus according to the manufacturer’s instructions. The sequences of shRNA against specific targets are available in Table S1.

### CircRNA sequencing

The total RNA of the cell samples was extracted using TRIzol reagent (Thermo Fisher Scientific, Waltham, MA, USA). The circRNA sequencing analysis was performed at Genechem (Shanghai, China) following the standard experimental procedures below.

#### RNA quantification and qualification

RNA degradation and contamination were monitored on 1% agarose gels. RNA purity was checked using the NanoPhotometer® spectrophotometer (IMPLEN, CA, USA). RNA concentration was measured using Qubit® RNA Assay Kit in Qubit® 2.0 Flurometer (Life Technologies, CA, USA). RNA integrity was assessed using the RNA Nano 6000 Assay Kit of the Bioanalyzer 2100 system (Agilent Technologies, CA, USA).

#### Library preparation for circRNA sequencing

A total of 5 μg RNA per sample was used as input material for the RNA sample preparations. Firstly, ribosomal RNA was removed by Epicentre Ribozero™ rRNA Removal Kit (Epicentre, USA), and the rRNA-free residue was cleaned up by ethanol precipitation. Subsequently, the linear RNA was digested with 3 U of RNase R (Epicentre, USA) per μg of RNA. Following the manufacturer’s recommendations, the sequencing libraries were generated by NEBNext® Ultra™ Directional RNA Library Prep Kit for Illumina® (NEB, USA). Briefly, fragmentation was carried out using divalent cations under elevated temperature in NEBNext First Strand Synthesis Reaction Buffer (5X). First-strand cDNA was synthesized using random hexamer primer and M-MuLV Reverse Transcriptase (RNaseH-). Second strand cDNA synthesis was performed using DNA Polymerase I and RNase H. In the reaction buffer, dNTPs with dTTP were replaced by dUTP. The remaining overhangs were converted into blunt ends via exonuclease/polymerase activities. After adenylation of 3′ ends of DNA fragments, NEBNext Adaptor with hairpin loop structure was ligated to prepare for hybridization. To select cDNA fragments of preferentially 150 ~ 200 bp in length, the library fragments were purified with the AMPure XP system (Beckman Coulter, Beverly, USA). Then 3 μl USER Enzyme (NEB, USA) was used with size-selected, adaptor-ligated cDNA at 37 °C for 15 min followed by 5 min at 95 °C before PCR. Then PCR was performed with Phusion High-Fidelity DNA polymerase, Universal PCR primers, and Index (X) Primer. At last, products were purified (AMPure XP system), and library quality was assessed on the Agilent Bioanalyzer 2100 system.

#### Clustering and sequencing

According to the manufacturer’s instructions, the clustering of the index-coded samples was performed on a cBot Cluster Generation System using TruSeq PE Cluster Kit v3-cBot-HS (Illumia). After cluster generation, the libraries were sequenced on an Illumina Hiseq 4000 platform, and 150 bp paired-end reads were generated.

### CircRNA sequencing-data analysis

Raw data (raw reads) of fastq format were firstly processed through in-house Perl scripts. In this step, clean data (clean reads) were obtained by removing reads containing adapter, ploy-N, and low-quality reads from raw data. At the same time, the clean data’s Q20, Q30, and GC content were calculated. All the downstream analyses were based on clean data with high quality. The genome and gene model annotation files were downloaded directly from the genome website. Index of the reference genome was built using Bowtie2 v2.2.8, and paired-end clean reads were aligned to the reference genome using Bowtie. The circRNAs were detected and identified using find_circ and CIRI2. Prior to differential gene expression analysis, the read counts were adjusted by edgeR program package for each sequenced library through one scaling normalized factor. Differential expression analysis was performed using the edgeR R package. Fold change > 2 or < 0.5 and *P*-value < 0.05 was set as the threshold for significantly differential expression.

### RNase R treatment

RNase R (Geneseed, Guangzhou, China) was applied to digest linear RNA. RNAs extracted from PANC-1-GR and BxPC-3-GR cells were divided into two groups for RNase R treatment and control. The sample was incubated for 30 min at 37 °C with 3 U/μg of RNase R. For analysis, qRT-PCR was used to detect the expression of MTHFD1L and circ-MTHFD1L. GAPDH in the control group was used as the internal reference. Three independent experiments were applied in triplicate.

### Actinomycin D (ActD) treatment

1 × 10^5^ cells per well of PANC-1-GR and BxPC-3-GR cells were seeded in a 6-well plate overnight, and 2 mg/L actinomycin D (Sigma, USA) was added into the well for 4, 8, 12, and 24 h. The cells were harvested according to the time of treatment. Then qPCR was performed to analyze the stability of MTHFD1L mRNA and circ-MTHFD1L. Three independent experiments were applied in triplicate.

### RNA extraction and quantitative real-time PCR (qRT-PCR) analysis

According to the manufacturer’s protocol, total RNA from PDAC cells, tissues, and matched non-cancerous tissues was isolated using the TRIzol Reagent (Thermo Fisher Scientific, Waltham, MA, USA). Reverse transcription was performed using the PrimeScript RT Reagent Kit (Takara, Dalian, China). Bulge-loop™ miRNA RT-qPCR Primers were applied to determine the level of miRNAs. The real-time PCR reactions were performed using StepOnePlus™ Real-Time PCR System (Thermo Fisher Scientific, MA, US). The program settings on temperature cycling were followed as instructed by the manufacturer. The relative circRNA/mRNA and miRNA expression levels were normalized to GAPDH and U6, respectively, using the 2^−△△CT^ method. The sequences of primers are listed in Table S1.

### Western blot analysis

In brief, proteins were isolated from PDAC cells and tumor tissues using RIPA buffer (Solarbio, Beijing, China) supplemented with proteinase inhibitors, and the protein concentration was determined with BCA reagent (Beyotime, Beijing, China). Cell lysates were separated on SDS-polyacrylamide gels and then transferred onto polyvinylidene difluoride (PVDF) membranes. (Millipore), After the membranes were blocked in 5% skim powdered milk for 2 h, they were incubated with primary antibodies overnight at 4 °C. The primary antibodies used in this study included: RPN6 (Cat. No. ER64693, HUABIO), RAD50 (Cat. No. sc-56,209, Santa Cruz), RAD51 (Cat. No. sc-398,587, Santa Cruz), BRCA1 (Cat. No. sc-6954, Santa Cruz), BRCA2 (Cat. No. sc-518,154, Santa Cruz), PCNA (Cat. No. EM111201, HUABIO), P53 (Cat. No. ET1605–16, HUABIO), P21 (Cat. No. 10355–1-AP, Proteintech), and GAPDH (Cat. No. ET1601–4, HUABIO). Next, the membranes were incubated with secondary antibodies (HUABIO, Hangzhou, China) at room temperature for 1 h. After washing three times, the targeted proteins were visualized using enhanced chemiluminescence (ECL) reagent (Millipore, MA, USA). GAPDH was used as the loading control in this study.

### Colony formation assay

Cells were seeded into 6-well plates at an initial density of 800 cells/well and grew for 24 h, then treated with gemcitabine at half the IC50 value of the respective cells for 48 h, after which the culture was continued for 10 days. The colonies were fixed in 4% paraformaldehyde and stained with 0.1% crystal violet (Sigma, St. Louis, MO, USA). The visible colonies were counted using a light microscope. The numbers of colonies in triplicate wells were measured for each treatment group.

### Cell proliferation assay

Cell proliferation was assessed by a Cell Counting Kit-8 (CCK-8) kit (Dojindo, Kumamoto, Japan). Cells (5 × 10^3^cells per well) were cultured in 96-well-plates for 0, 24, 48, 72, and 96 h, and treated with gemcitabine at the IC50 value of the respective cells, after which CCK-8 solution (10 μl) was added to each well, and the plates were further incubated for 2 h. The number of cells was quantified by measuring the absorbance at 450 nm on a microplate reader. Three independent experiments were performed. The sensitivity to gemcitabine of pancreatic cancer cells was determined using the CCK-8 assay.

### Cytotoxicity assay

Briefly, Cells (5 × 10^3^cells per well) were seeded onto 96-well plates and incubated at 37 °C overnight. Cells were then treated with different concentrations of gemcitabine. After incubation for 48 h, CCK-8 solution (10 μl) was added to each well, and the plates were further incubated for 2 h. The number of cells was quantified by measuring the absorbance at 450 nm on a microplate reader. Dose-response curves were plotted on a semilog scale as the percentage of the control cell number, which was obtained from the sample with no drug exposure. The IC50 was determined by the intersection of the gemcitabine concentration and the midpoint of the 450 nm reading.

### Cell cycle assay

A cell cycle assay kit (Cat. no. C1052) was purchased from Beyotime Institute of Biotechnology (Haimen, China). The drug-resistant cells were treated with gemcitabine at half the respective IC50 concentrations for 24 h, and then cells were fixed with 70% ethanol at 4 °C for 24 h and then resuspended in ice-cold PBS before they were resuspended with a staining solution containing RNase A and propidium iodide (PI). The DNA content was assessed using a flow cytometer, and the number of cells in the different cell cycle phases was counted using ModFit version 5.0 (Verity Software House, Topsham, ME, USA). Three independent experiments were performed.

### Fluorescence in situ hybridization (FISH)

The specific fluorescently labeled circ-MTHFD1L and miR-615-3p FISH probes were designed and synthesized by Servicebio (Wuhan, China). The FISH experiment was performed according to the manufacturer’s instructions. All images were acquired on Nikon A1Si Laser Scanning confocal microscope (Nikon Instruments Inc., Japan). The sequences of probes are listed in Table S1.

### Dual-luciferase reporter assay

The full-length wild-type (WT) sequence of circ-MTHFD1L, the RPN6 3′ untranslated region (UTR), and the indicated mutant circ-MTHFD1L and RPN6 3’UTR (Mut) containing the predicted miR-615-3p binding sites were separately synthesized and cloned into the dual-luciferase reporter vector PGL3 (Genechem, Shanghai, China). The resulting dual-luciferase reporter plasmids (WT or Mut) were co-transfected with the miR-615-3p mimic or inhibitor into PANC-1-GR or BxPC-3-GR cells, respectively, using Lipofectamine 3000. After 48 h of incubation, the relative firefly luciferase activities concerning the corresponding Renilla luciferase activities were measured and analyzed using a Dual-Luciferase Assay System (Promega, Fitchburg, WI, USA) following the manufacturer’s protocol.

### RNA pull-down assay

A biotin-labeled probe targeting circ-MTHFD1L and a random oligo probe (RiboBio, Guangzhou, China) were incubated with M280 streptavidin dynabeads (Invitrogen, USA) at 25 °C for 2 h to generate probe-coated beads. The cell lysates were incubated with the probe-coated beads mixture at 4 °C overnight. Subsequently, the circ-MTHFD1L/miRNA/bead complexes were washed three times and eluted from the beads. Then, the enrichment of circ-MTHFD1L and related miRNAs in the precipitated complexes was evaluated by qRT-PCR. The probe sequence of RNA pull-down is shown in Table S1.

### RNA immunoprecipitation (RIP)

RNA immunoprecipitation (RIP) assay was performed using Magna RIP™ RNA-binding protein immunoprecipitation kit (Millipore, Billerica, MA, USA) following the manufacturer’s protocol. Transfected cells were lysed in complete RNA immunoprecipitation lysis buffer after transfected with miR-615-3p mimics or negative control. Then, the cell extract was incubated with magnetic beads conjugated with anti-Argonaute 2 (AGO2) or anti-IgG antibody (Millipore, Billerica, MA, USA) for 6 h at 4 °C. The beads were washed and incubated with Proteinase K to remove proteins. Finally, isolated RNA was extracted using TRIzol Reagent (Thermo Fisher Scientific, Waltham, MA, USA), then the purified RNA was subjected to agarose gel electrophoresis and qRT-PCR analysis.

### Neutral comet assay

Neutral comet assays were performed using an OxiSelect Comet Assay Kit (Cell Biolabs, USA) per the manufacturer’s protocol. Briefly, cells were dissociated with Accutase and washed with PBS, and replicates were suspended in OxiSelect comet agarose (Cell Biolabs, USA). Neutral electrophoresis was conducted at 30 V for 30 min. Data were collected with a fluorescent microscope with a FITC filter and analyzed using the OpenComet software [24]. All steps after agarose treatment were conducted in the dark to prevent additional DNA damage.

### Animal experiments

4–6 week-old male athymic BALB/c nude mice (Slac Laboratory Animal Co. Ltd., Shanghai, China) were housed and fed in standard pathogen-free conditions. For the subcutaneous cell line-derived xenograft (CDX) model, Gemcitabine-resistant PANC-1 cells (PANC-1-GR) were prepared as stable gene expression cell lines after transduction with sh-NC or sh-circ-MTHFD1L, vector or circ-MTHFD1L. Subcutaneous injections of 5 × 10^6^ cells in 100 μL serum-free DMEM and Matrigel (1:1) were performed on each nude mouse (*n* = 6 in each group). All surgeries were performed under sodium pentobarbital anesthesia, and all efforts were made to minimize suffering. Gemcitabine was suspended in an oral vehicle containing Cremophor (Sigma-Aldrich), 95% ethanol, and water in a ratio of 1:1:6. Four weeks later, the mice were treated with gemcitabine (50 mg/kg/mouse twice a week by tail vein injection). The mice were sacrificed after 4 weeks of treatment. For two generations of the subcutaneous gemcitabine-resistant CDX model, PANC-1-GR cells were transplanted subcutaneously into the axilla of 4–6 week-old male BALB/c nude mice (Slac Laboratory Animal Co. Ltd., Shanghai, China). After 4 weeks, all mice were treated with gemcitabine (50 mg/kg/mouse twice a week by tail vein injection). 10 weeks later, the most resistant xenograft was isolated and mechanically disaggregated into approximately 1–2 mm^3^ tissue blocks to implanted subcutaneously into the axilla of 4–6 week-old male BALB/c nude mice for the second CDX generation. Two weeks after implantation, all mice were treated with sh-circ-MTHFD1L lentivirus (twice a week) and randomized into four groups (*n* = 6 mice per group). The four groups were treated as follows: gemcitabine alone, olaparib alone, gemcitabine combined with olaparib, and negative control. The mice were euthanized in the sixth week, and tumors were isolated for further studies. The subcutaneous tumor size was measured and recorded every 2 days using the Vernier caliper: tumor volume (mm^3^) = (L × W^2^)/2, where L is the long axis, and W is the short axis. All the animal experiments were approved by the Animal Welfare Committee of Fujian Medical University (Fuzhou, China).

### Bioinformatics analysis

The public datasets GSE79634, GSE110580, GSE112264, and GSE113486, were retrieved from the Gene Expression Omnibus (GEO, https://www.ncbi.nlm.nih.gov/geo/) database. Differential expression analysis was performed using the limma R package with Fold change > 2 or < 0.5 and *P*-value < 0.05 as the screening criterion. The downstream miRNAs of circ-MTHFD1L were predicted through ENCORI (ClipExpNum ≥1, https://starbase.sysu.edu.cn/), CircInteractome (https://circinteractome.irp.nia.nih.gov/), Circbank (MiRanda binding site ≥1 & targetscan binding site ≥2, http://www.circbank.cn/) and CircAtlas (http://circatlas.biols.ac.cn/), and then intersected miRNAs served as a candidate miRNA. The downstream target genes of miR-615-3p were predicted through ENCORI (ClipExpNum ≥1, https://starbase.sysu.edu.cn/), miRDB (Target Score ≥ 50, http://mirdb.org/), mirDIP (Integrated score > 0.266, http://ophid.utoronto.ca/mirDIP/), miRTarBase (Sum ≥1, https://miRTarBase.cuhk.edu.cn/) and TargetScan (Conserved sites ≥1, http://www.targetscan.org/), and then intersected with the downstream target genes of miR-615-3p to identify the candidate gene. The expressions of MTHFD1L and RPN6 in pancreatic cancer and normal pancreatic tissue from TCGA and GTEx databases and the survival curves of MTHFD1L and RPN6 in TCGA database were obtained by GEPIA2 (http://gepia2.cancer-pku.cn/).

### Statistical analysis

GraphPad Prism 8.0 and SPSS 23.0 were applied for statistical analysis. Differences between the indicated groups were compared using the Student’s t-test and one-way analysis of variance (ANOVA) followed by Fisher’s least significant difference (LSD) test. Correlations were evaluated by Pearson correlation analysis. The cumulative overall survival (OS) and progression-free survival (PFS) rates were calculated using the Kaplan-Meier method, and significance was evaluated with the log-rank test. A *P*-value < 0.05 was considered to indicate a statistically significant result.

## Result

### Discovery and characteristics of a new gemcitabine resistance-related circRNA in pancreatic cancer

**Fig. 1:**
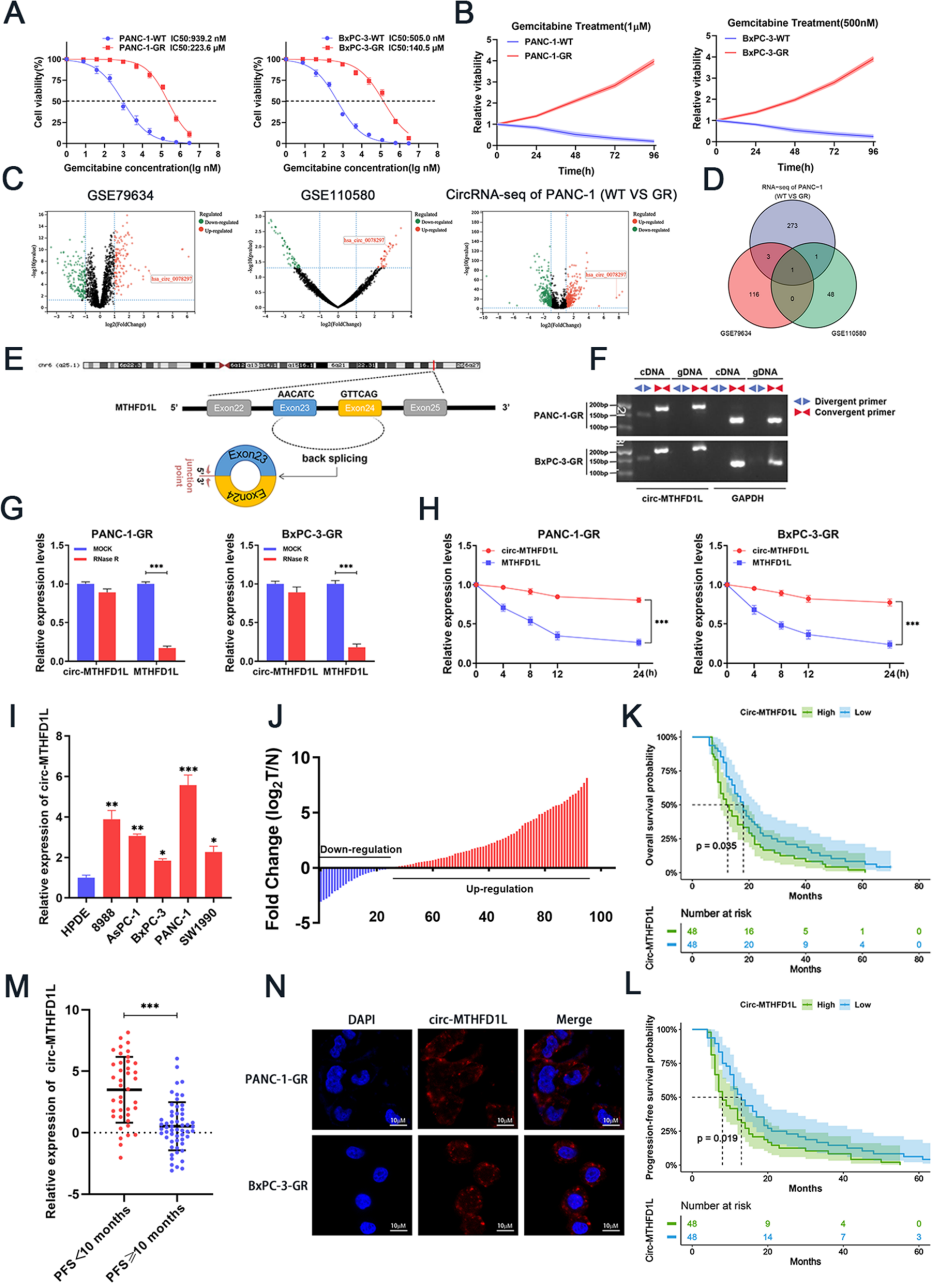
Discovery and characteristics of a new gemcitabine resistance-related circRNA in pancreatic cancer. **A**-**B** Two gemcitabine-resistant PDAC cell lines (PANC-1-GR, BxPC-3-GR) were established and confirmed using the IC50 and Cell viability **C** Volcano plots showing the up-regulated (red) and down-regulated (blue) circRNAs in PDAC from GSE79634, GSE69362 and circRNA-seq of PANC-1 (WT vs GR) (fold change > 2 or < 0.5, *P <* 0.05). **D** The Venn diagram shows the intersection of upregulated circRNA among the three results. **E** Schematic diagram of chromosomal location and formation of circ-MTHFD1L. **F** PCR detection of circ-MTHFD1L and linear transcript of MTHFD1L by divergent and convergent primers in cDNA and genomic DNA (gDNA). **G** Relative RNA level of circ-MTHFD1L and linear MTHFD1L treated with RNase R. **H** Relative RNA level of circ-MTHFD1L and linear MTHFD1L treated with actinomycin D at the indicated time. **I** The expression levels of circ-MTHFD1L in PADC cells (8988, AsPC-1, BxPC-3, PANC-1, SW1990) and normal human pancreatic duct epithelial (HPDE) were analyzed by qRT-PCR. **J** The expression levels of circ-MTHFD1L in tumor and matched non-tumor tissues from 96 gemcitabine-treated PADC patients were analyzed by qRT-PCR. **K-L** Kaplan-Meier survival analysis showed that PADC patients with low and high circ-MTHFD1L expression of overall survival and PFS. The median circ-MTHFD1L expression was used as the cutoff value. **M** Relative RNA level of circ-MTHFD1L in PFS < 10 months group and PFS ≥ 10 months group were analyzed by qRT-PCR. **N** Localization of circ-MTHFD1L (red) in PANC-1-GR cells and BxPC-3-GR cells using fluorescence in situ hybridization(FISH). Cell nuclei were counterstained with DAPI (blue). Scale bar, 50 μm. Data are shown as mean ± SD. *P < 0.05; ** *P <* 0.01; *** *P <* 0.001, between the indicated groups

To discover potential circRNAs related to chemotherapy resistance of pancreatic cancer, we constructed two gemcitabine resistant cell lines (PANC-1-GR, BxPC-3-GR) and evaluated their resistance by detecting cell viability. As shown in Fig. 1A-B, the IC50 level of gemcitabine resistant strains was much higher than that of wild-type cells, and gemcitabine resistant strains showed better cell viability under the intervention of gemcitabine. Next, we performed circRNA sequencing of PANC-1 gemcitabine resistant cells and their wild-type cells and analyzed the differential expression of circRNAs. At the same time, we analyzed the Gene Expression Omnibus (GEO) dataset GSE79634 (20 pairs of pancreatic cancer tissues and corresponding paracancerous tissues.) and GSE110580 (3 pairs of PANC-1 gemcitabine-resistant cells and their wild-type cells) [25, 26]. The volcano plot shows the differentially expressed circRNAs in the three sets of data (screening criteria: fold change > 2 or < 0.5 and *P* < 0.05) (Fig. 1C). We focused on circRNA, significantly upregulated in pancreatic cancer and PANC-1 gemcitabine-resistant cells. The Venn diagram showed only one circRNA: hsa_circ_0078297 (unpublished data), upregulated in three datasets (Fig. 1D). CircBase and UCSC Genome Browser database show that hsa_circ_0078297 is produced by reverse splicing of the 23rd and 24th exons of the human MTHFD1L gene on chromosome 6q25.1, which contains 279 nucleotides (Fig. 1E). Therefore, we named hsa_circ_0078297 circ-MTHFD1L.

To clarify the circular characteristics of circ-MTHFD1L, we designed divergent and convergent primers. The PCR results showed that circ-MTHFD1L could be amplified by the divergent primers of PANC-1-GR and BxPC-3-GR cells instead of gDNA. Linear transcripts can be amplified by convergent primers (Fig. 1F). To verify the stability of circ-MTHFD1L, we treated the extracted RNA with RNase R (linear RNA degradation product) and found that circ-MTHFD1L has obvious resistance to RNase R in PANC-1-GR and BxPC-3-GR cells (Fig. 1G). In addition, after treating the cells with ActD (a transcription inhibitor), we found that circ-MTHFD1L is more stable than linear MTHFD1L (Fig. 1H).

Next, we tested the expression level of circ-MTHFD1L in human pancreatic cancer cell lines (8988, AsPC-1, BxPC-3, PANC-1, SW1990) and normal human pancreatic duct epithelial cells (HPDE), and we found Compared with normal human pancreatic duct epithelial cells, circ-MTHFD1L was significantly upregulated in pancreatic cancer cells (Fig. 1I). To further study the prognostic significance of circ-MTHFD1L expression in pancreatic cancer patients, we tested the expression of circ-MTHFD1L in 96 pairs of pancreatic cancer tissues and normal tissues from gemcitabine-treated PADC patients. The results showed that the expression of circ-MTHFD1L was significantly upregulated in tumor tissues compared with matched normal tissues (Fig. 1J). Kaplan-Meier survival analysis showed that patients with higher expression levels of circ-MTHFD1L were associated with worse overall survival (OS) and progression-free survival (PFS) (Fig. 1K, L). The PFS is often used as an important index of drug efficacy internationally. Therefore, we divided the patients into two groups based on the median PFS (10 months): PFS < 10 months group (faster progression after gemcitabine treatment) and PFS ≥ 10 months group (slower progression after gemcitabine treatment). After further analysis of qRT-PCR results, we found that circ-MTHFD1L expression was higher in patients with PFS < 10 months than patients with PFS ≥ 10 months (Fig. 1M). We also analyzed the correlation of circ-MTHFD1L expression with clinicopathological characteristics of PDAC patients and the correlation of different PFS groups with circ-MTHFD1L expression and clinicopathological characteristics of PDAC patients, we found that circ-MTHFD1L expression increased with poor differentiation and elevated CA19–9 level (Table S2), and worse PFS was associated with high expression of circ-MTHFD1L (Table S3). In addition, fluorescence in situ hybridization (FISH) showed that circ-MTHFD1L is mainly located in the cytoplasm (Fig. 1N). In summary, we found a potential circRNA, circ-MTHFD1L, associated with gemcitabine resistance in pancreatic cancer. Its upregulation indicates a poor prognosis and is mainly located in the cytoplasm.

### Circ-MTHFD1L is crucial for maintaining gemcitabine resistance

**Fig. 2:**
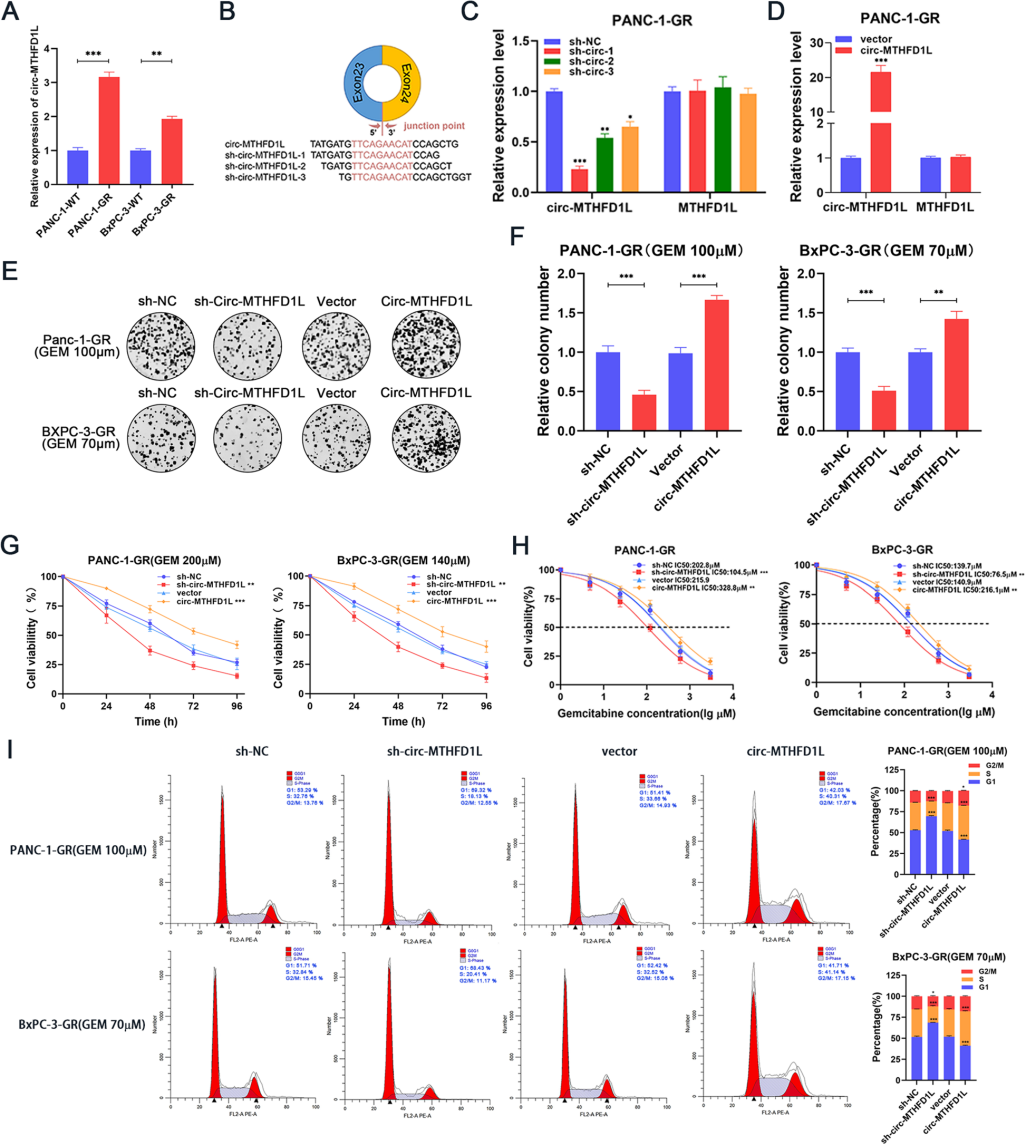
Circ-MTHFD1L is crucial for maintaining gemcitabine resistance. **A** Quantitative RT-PCR (qRT-PCR) analysis of circ-MTHFD1L in the two gemcitabine-resistant cell lines compared with their parental cells. **B** Three knockdown sequences were designed to target the circ-MTHFD1L junction site **C**-**D** The overexpression and knockdown efficiency of circ-MTHFD1L were confirmed by qRT-PCR analysis of circ-MTHFD1L and MTHFD1L mRNA in PANC-1-GR. **E**-**H** The proliferative ability of stably transfected PANC-1-GR or BxPC-3-GR cells dealing with gemcitabine was investigated via colony formation assays (**E**, **F**) and CCK-8 assays (cell viability and IC50) (**G**, **H**). Representative colony formation images are shown (**E**), and the numbers of colonies were summarized (**F**). **I** Flow cytometry analysis of the cell cycle progression of stably transfected PANC-1-GR or BxPC-3-GR cells dealing with gemcitabine was performed. Representative images and quantification of the results are presented. Data are shown as mean ± SD. *P < 0.05; **P < 0.01; ***P < 0.001, between the indicated groups

The qRT-PCR analysis confirmed that the expression of circ-MTHFD1L in the two gemcitabine-resistant cell lines was significantly higher than that of its wild-type cells (Fig. 2A), while the parental gene MTHFD1L mRNA did not change significantly (Fig. S1A). In addition, the GEPIA2 database shows that the expression level of MTHFD1L mRNA does not correlate with the prognosis of patients (Fig. S1B). Therefore, circ-MTHFD1L may exert its function independently of the parental gene MTHFD1L mRNA. To explore the role of circ-MTHFD1L in gemcitabine resistance, we designed three knockdown sequences (Fig. 2B) for the junction site of circ-MTHFD1L and transduced them to PANC-1-GR and BxPC-3-GR through lentivirus. The qRT-PCR results showed that the knockdown effect of sh-circ-MTHFD1L-1 was the most significant, and it did not affect the expression of MTHFD1L mRNA (Fig. 2C and Fig. S1C). Therefore, we use the sh-circ-MTHFD1L-1 cell line in our subsequent experiments. In addition, we also stably overexpress circ-MTHFD1L in PANC-1-GR and BxPC-3-GR through lentivirus-mediated transduction (Fig. 2D and Fig. S1D). Next, we studied the effect of circ-MTHFD1L on the drug resistance of two pancreatic cancer cell lines resistant to gemcitabine. Colony formation assays showed that the number of colonies was significantly reduced after circ-MTHFD1L knockdown under gemcitabine loading, while significantly increased after circ-MTHFD1L overexpressed (Fig. 2E, F). Cell viability testing produced similar results (Fig. 2G). To further confirm the effect of circ-MTHFD1L on gemcitabine resistance, we tested the IC50 value after knockout and overexpression of circ-MTHFD1L in gemcitabine-resistant cell lines. We found that after knockdown of circ-MTHFD1L, The IC50 values of PANC-1-GR and BxPC-3-GR cells were significantly reduced and significantly increased after circ-MTHFD1L overexpression (Fig. 2H). Gemcitabine is a cell cycle-specific drug that mainly acts on cells in the DNA synthesis phase, S phase cells, and can block development from the G1 to S phase [27, 28]. To explore the effect of circ-MTHFD1L on the progression of the cell cycle under gemcitabine load, we performed flow cytometry analysis. The results showed that circ-MTHFD1L knockdown significantly reduced the proportion of cells in the S phase, while overexpression of circ-MTHFD1L induced The opposite cell cycle changes (Fig. 2I). The above experiments show that circ-MTHFD1L is very important for maintaining gemcitabine resistance.

### Circ-MTHFD1L acts as a miRNA sponge for miR-615-3p

**Fig. 3:**
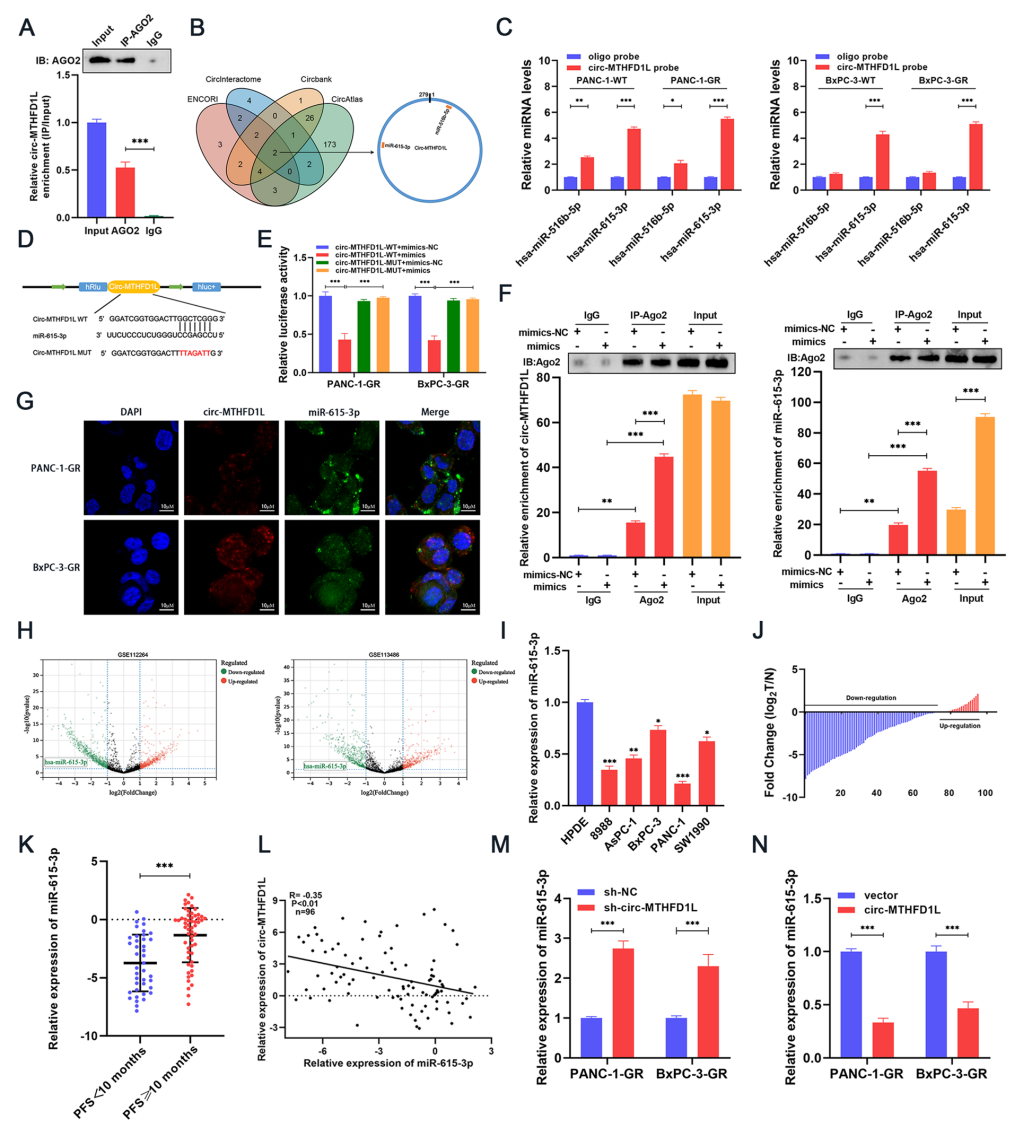
Circ-MTHFD1L acts as a miRNA sponge for miR-615-3p. **A** RIP assay showed the association between AGO2 and circ-MTHFD1L. Top, IP efficiency of the anti-AGO2 antibody in Western blots. Bottom, relative enrichment represents RNA associated with AGO2 relative to the input control. An IgG antibody served as a control. **B** The potential target miRNAs of circ-MTHFD1L were predicted in the ENCORI, circInteractome, circbank, and circAtlas databases. **C** QRT-PCR was utilized to determine the relative expression levels of miR-516b-5p and miR-615-3p in precipitates from PANC-1-GR and BxPC-3-GR cell lysate pulled down by the circ-MTHFD1L probe or oligo probe. **D** Schematic diagram of the circ-MTHFD1L-WT and circ-MTHFD1L-Mut luciferase vectors. **E** Relative luciferase activities in PANC-1-GR and BxPC-3-GR cells co-transfected with circ-MTHFD1L-WT or circ-MTHFD1L-Mut miR-615-3p mimic, inhibitor, or corresponding negative control. **F** RIP assay was carried out with anti-AGO2 antibodies or IgG in PANC-1-GR cells after transfection with the miR-615-3p mimic or mimic NC, and qRT-PCR was then performed to detect the enrichment of circ-MTHFD1L and miR-615-3p. **G** The co-localization of circ-MTHFD1L (red) and miR-615-3p (green) was observed by fluorescence in situ hybridization (FISH) in PANC-1-GR cells. Cell nuclei were counterstained with DAPI (blue). Scale bar: 50 μm. **H** Volcano plots showing the up-regulated (red) and down-regulated (blue) miRNAs in PDAC from GSE112264*,* GSE113486 (fold change > 2 or < 0.5, *P <* 0.05). **I** The expression levels of miR-615-3p in PADC cells (8988, AsPC-1*,* BxPC-3*,* PANC-1*,* SW1990) and normal human pancreatic duct epithelial (HPDE) were analyzed by qRT-PCR. **J** The expression levels of circ-MTHFD1L in tumor and matched non-tumor tissues from 96 gemcitabine-treated PADC patients were analyzed by qRT-PCR. **K** Relative RNA level of circ-MTHFD1L in PFS < 10 months group and PFS ≥ 10 months group were analyzed by qRT-PCR. **L** The correlation between circ-MTHFD1L and miR-615-3p in 96 gemcitabine-treated PDAC patients was analyzed by Pearson correlation analysis. **M-N** The relative expression of miR-615-3p in PANC-1-GR or BxPC-3-GR cells was analyzed by qRT-PCR after indicated transfection. Data are shown as mean ± SD. *P < 0.05; **P < 0.01; ***P < 0.001, between the indicated groups

Previous studies have shown that circRNA can act as a miRNA sponge and regulate the expression of downstream genes by adsorbing miRNA [29]. We predicted through CSCD and circatlas databases that circ-MTHFD1L may bind to AGO2 (Fig. S2A, B), and given that circ-MTHFD1L is located in the cytoplasm, we hypothesized that miRNA sponge activity might be a possible mechanism of its function. We performed a RIP test with AGO2 antibody in PANC-1-GR cell lysate to verify this hypothesis. We were able to detect circ-MTHFD1L (Fig. 3A) in AGO2 immunoprecipitated RNA, indicating that circ-MTHFD1L can bind to miRNA through AGO2 protein. Subsequently, we used four databases (ENCORI, CircInteractome, Circbank, CircAtlas) to predict the miRNA that circ-MTHFD1L might bind to by bioinformatics. In the intersection of these 4 databases, we screened out 2 candidate miRNAs (Fig. 3B, Table S4). Next, we used the biotin-labeled circ-MTHFD1L probe to perform RNA pull-down experiments in PANC-1-GR and BxPC-3-GR cells. The PCR results showed that miR-615-3p could be significantly enriched in PANC-1-GR and BxPC-3-GR cells (Fig. 3C). To further verify the binding of circ-MTHFD1L and miR-615-3p, we constructed two circ-MTHFD1L luciferase reporter plasmids: wild type and miR-615-3p binding site mutant (Fig. 3D). The luciferase activity of wild type can be significantly inhibited by miR-615-3p mimics, but the luciferase activity of mutant has no significant change (Fig. 3E). Next, we performed anti-ago2-RIP experiments in PANC-1-GR cells. Compared with IgG, circ-MTHFD1L and miR-615-3p were significantly pulled down by the ago2 antibody. Compared with the mimic-NC group, transfected miR-615-3p mimics cells are more abundant in circ-MTHFD1L and miR-615-3p (Fig. 3F). In addition, FISH results found that both circ-MTHFD1L and miR-615-3p were co-localized in the cytoplasm of PANC-1-GR cells (Fig. 3G). The above experiments confirmed that circ-MTHFD1L specifically binds to miR-615-3p. Next, through the bioinformatics analysis of the public data sets GSE112264 and GSE113486, it was found that miR-615-3p is relatively low in the serum of patients with pancreatic cancer (Fig. 3H). We tested the expression level of miR-615-3p in human pancreatic cancer cell lines, and we found that compared with normal human pancreatic duct epithelial cells, miR-615-3p was significantly down-regulated in pancreatic cancer cells (Fig. 3I). In addition, we detected the expression of miR-615-3p in tumor tissues and normal tissues of 96 gemcitabine-treated PADC patients. The results showed that the expression of miR-615-3p in tumor tissues was significantly down-regulated compared with matched normal tissues (Fig. 3J), and the expression of miR-615-3p was lower in PFS < 10 months compared to PFS ≥ 10 months (Fig. 3K). Correlation analysis shows that the expression of miR-615-3p is negatively correlated with the expression of circ-MTHFD1L (Fig. 3L). We further found that the overexpression of circ-MTHFD1L significantly reduced the expression of miR-615-3p, while the knockout of circ-MTHFD1L significantly increased its expression (Fig. 3M, N). In summary, circ-MTHFD1L can act as a miRNA sponge for miR-615-3p and negatively regulate miR-615-3p.

### MiR-615-3p reverses gemcitabine resistance of circ-MTHFD1L

**Fig. 4:**
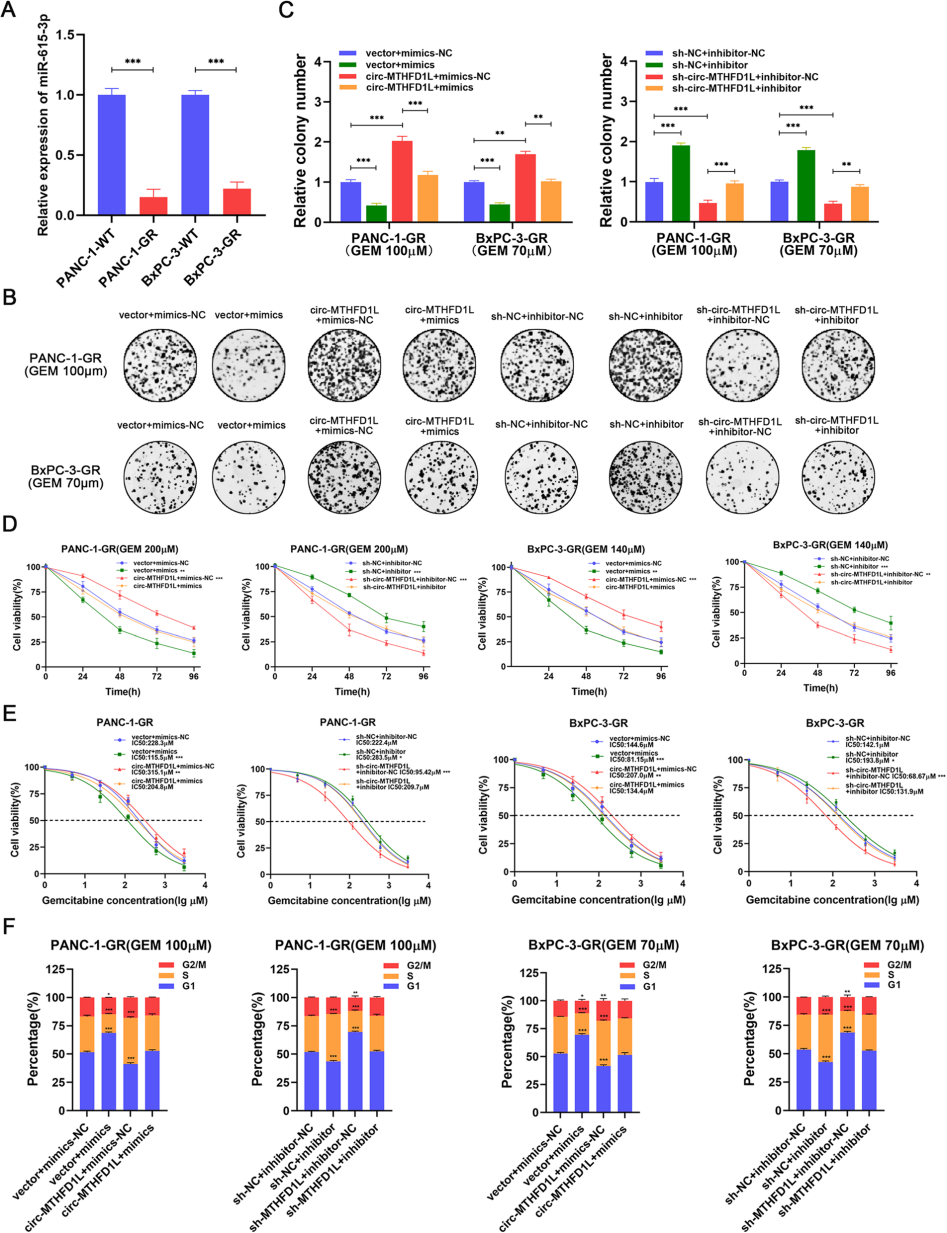
MiR-615-3p reverses gemcitabine resistance of circ-MTHFD1L. **A** Quantitative RT-PCR (qRT-PCR) analysis of miR-615-3p in the two gemcitabine-resistant cell lines compared with their parental cells. **B-E** The proliferative ability of the indicated cells dealing with gemcitabine was investigated via colony formation assays (**B**, **C**) and CCK-8 assays (cell viability and IC50) (**D**, **E**). Representative colony formation images are shown (**B**), and the numbers of colonies were summarized (**C**). **F** Flow cytometry analysis of the cell cycle progression of the indicated cells dealing with gemcitabine was performed. Representative images and quantification of the results are presented. Data are shown as mean ± SD. *P < 0.05; **P < 0.01; ***P < 0.001, between the indicated groups

Through qRT-PCR analysis, we found that the expression of miR-615-3p in PANC-1-GR and BxPC-3-GR were significantly lower than their wild-type cells (Fig. 4A). To clarify whether circ-MTHFD1L affects gemcitabine resistance through miR-615-3p, we constructed cells for rescue experiments and verified the transfection efficiency by qRT-PCR (Fig. S3A-D). Then we performed rescue experiments. Colony formation assays showed that overexpression of miR-615-3p significantly reduced the number of colonies and reversed the cellular phenotype of circ-MTHFD1L, while inhibition of miR-615-3p significantly increased the number of colonies and rescued sh-circ-MTHFD1L cell phenotype (Fig. 4B, C). Cell viability testing also produced similar results (Fig. 4D). Then we further clarified the effect of miR-615-3p on gemcitabine resistance by testing the IC50 value. Overexpression of miR-615-3p reduced the IC50 value of gemcitabine resistant cell lines and reversed the gemcitabine resistance of circ-MTHFD1L. Inhibition of miR-615-3p showed the opposite result (Fig. 4E). In addition, we found through flow cytometry that miR-615-3p can significantly reduce the proportion of cells in the S phase and reverse the effect of circ-MTHFD1L on the cell cycle under gemcitabine loading (Fig. 4F). In summary, miR-615-3p can increase the sensitivity of gemcitabine and reverse the biological effects of circ-MTHFD1L.

### The target gene of miR-615-3p, RPN6, is upregulated in pancreatic cancer and is associated with a poor prognosis

**Fig. 5:**
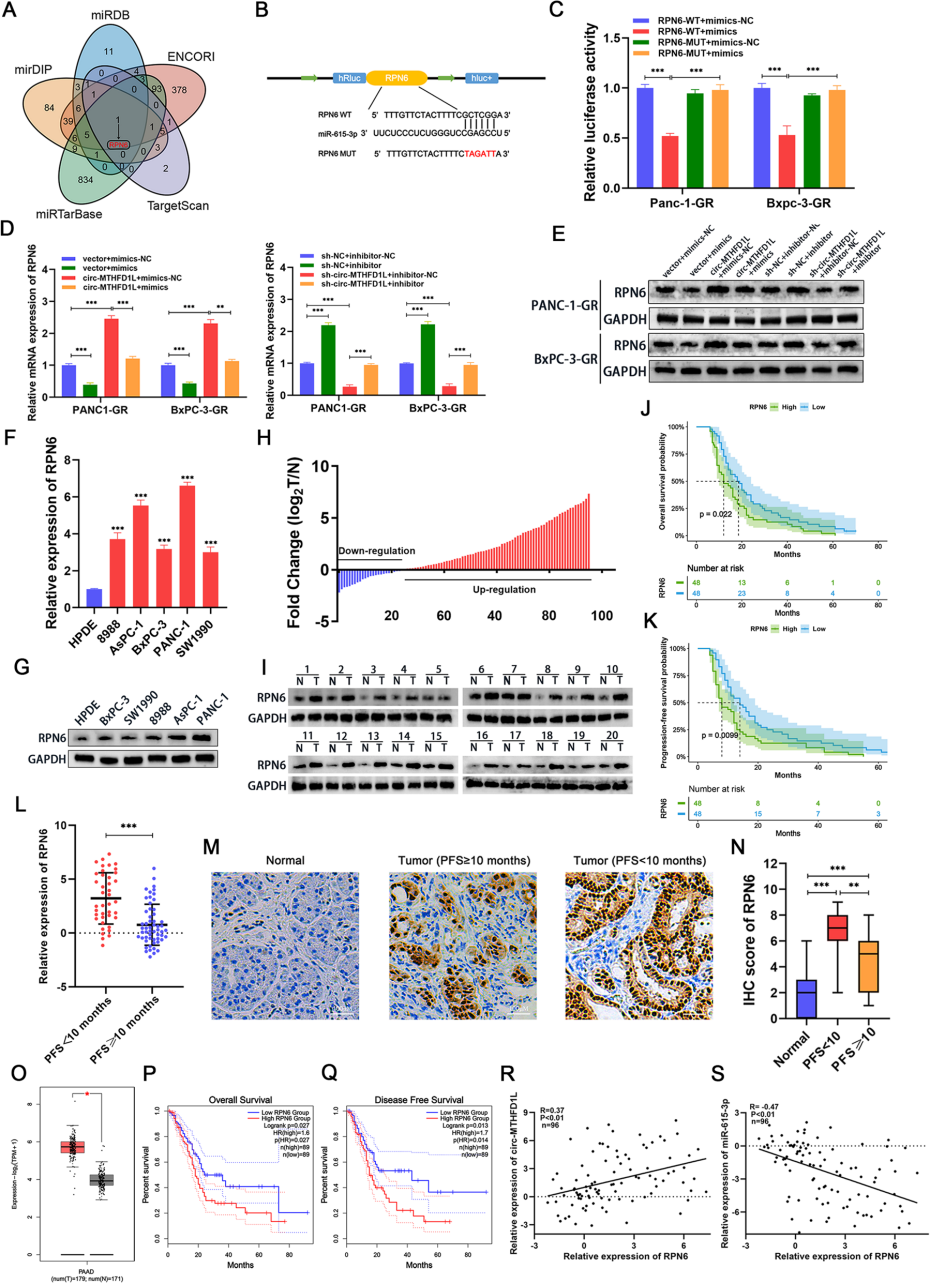
The target gene of miR-615-3p, RPN6, is upregulated in pancreatic cancer and is associated with a poor prognosis. **A** The potential target mRNAs of miR-615-3p were predicted in ENCORI, miRDB, mirDIP, miRTarBase, and TargetScan databases. **B** Schematic diagram of the RPN6-WT and RPN6-Mut luciferase vectors. **C** Relative luciferase activities in PANC-1-GR and BxPC-3-GR cells co-transfected with RPN6-WT or RPN6-Mut and the miR-615-3p mimic, inhibitor or corresponding negative control. **D**-**E** The relative expression of RPN6 in PANC-1-GR or BxPC-3-GR cells was analyzed by qRT-PCR and western blot after indicated transfection. **F-G** The expression levels of RPN6 in PADC cells (8988*,* AsPC-1*,* BxPC-3*,* PANC-1*,* SW1990) and normal human pancreatic duct epithelial (HPDE) were analyzed by qRT-PCR and western blot assays. **H-I** The expression levels of RPN6 in tumor and matched non-tumor tissues from 96 gemcitabine-treated PADC patients were analyzed by qRT-PCR (**H**). There are representative images of RPN6 protein levels in PDAC tumors and adjacent normal tissues by western blot assays (**I**). **J-K** Kaplan-Meier survival analysis showed that PADC patients with low and high circ-MTHFD1L expression of overall survival and PFS. The median circ-MTHFD1L expression was used as the cutoff value. **L** Relative RNA level of RPN6 in PFS < 10 months group and PFS ≥ 10 months group. **M**-**N** IHC staining shows the abundances of RPN6 in the indicated groups (**M**). The IHC scores of RPN6 were further quantified (**N**). **O** Box-plot of RPN6 expression in TCGA PDAC tumor and matched TCGA normal pancreatic tissues along with GTEx data. **P-Q** Overall survival and DFS of PDAC patients (*N* = 178) from TCGA project with high or low RPN6 expression levels. **R**-**S** The correlation between RPN6 and circ-MTHFD1L and miR-615-3p in PDAC was analyzed by Pearson correlation analysis. Data are shown as mean ± SD. *P < 0.05; **P < 0.01; ***P < 0.001, between the indicated groups

To further study the downstream target genes of miR-615-3p, we used five databases (ENCORI, miRDB, mirDIP, miRTarBase, TargetScan) to predict the mRNA that miR-615-3p-binding mRNA. We screened only one downstream target gene in the intersection of five databases: RPN6 (Fig. 5A, Table S5). To verify the binding relationship between RPN6 and miR-615-3p, we constructed two RPN6 luciferase reporter plasmids: wild type and miR-615-3p binding site mutant (Fig. 5B). The luciferase activity of wild type can be significantly inhibited by miR-615-3p mimics, while the luciferase activity of mutant has no significant change (Fig. 5C). In addition, the overexpression of miR-615-3p significantly reduced the expression of RPN6 and reversed the promotion of RPN6 after Circ-MTHFD1L overexpression. At the same time, miR-615 -3p inhibition significantly increased the expression of RPN6 and restored the inhibitory effect of circ-MTHFD1L knockdown on RPN6 expression, which was confirmed by qRT-PCR and western blotting (Fig. 5D, E). Next, we tested the expression level of RPN6 in human pancreatic cancer cell lines by qRT-PCR and western blotting. Compared with normal human pancreatic duct epithelial cells, RPN6 was significantly upregulated in pancreatic cancer cells (Fig. 5F, G). Then we tested the expression of RPN6 in 96 pairs of pancreatic cancer tissues and normal tissues from gemcitabine-treated PADC patients. The results showed that the expression of RPN6 was significantly upregulated in tumor tissues compared with matched normal tissues (Fig. 5H, I). Kaplan-Meier survival analysis showed that patients with higher expression levels of RPN6 were associated with worse overall survival (OS) and progression-free survival (PFS) (Fig. 5J, K), and the expression of RPN6 was higher in PFS < 10 months compared to PFS ≥ 10 months (Fig. 5L). We also analyzed the correlation of different PFS groups with RPN6 expression, and we found worse PFS was associated with high expression of RPN6 (Table S3). We further confirmed that RPN6 was highly expressed in cancer tissues and more highly expressed in the worse PFS group (PFS < 10 months) by immunohistochemistry (Fig. 5M, N). In addition, the GEPIA2 database confirmed that the expression of RPN6 in pancreatic cancer tissues was significantly increased (Fig. 5O). More importantly, compared with patients with lower RPN6 expression, patients with higher RPN6 expression have worse overall survival and disease-free survival (Fig. 5P, Q). Correlation analysis shows that the expression of circ-MTHFD1L was positively correlated with RPN6 (Fig. 5R) while the expression of miR-615-3p was negatively correlated with the expression of RPN6 (Fig. 5S). In summary, RPN6 is a downstream target gene of miR-615-3p, and its high expression is closely related to the worse prognosis of PDAC patients.

### RPN6 enhances gemcitabine resistance by promoting cell cycle progression and DNA damage repair

**Fig. 6:**
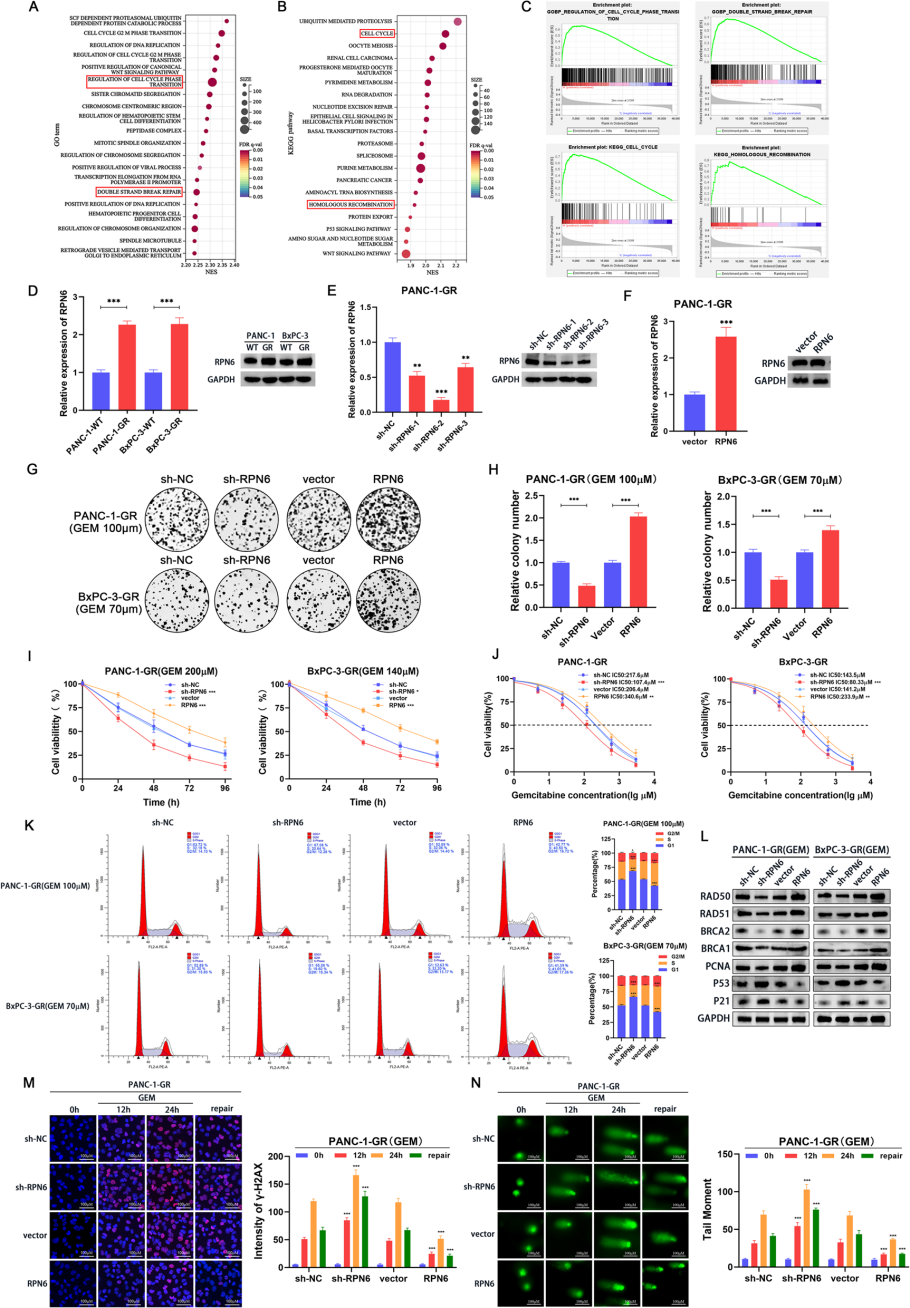
RPN6 enhances gemcitabine resistance by promoting cell cycle progression and DNA damage repair. **A-B** The bubble chart shows the significant enrichment of Gene Ontology (GO) term and KEGG pathway via Gene Set Enrichment Analysis (GSEA) grouped by RPN6 expression level based on RNA-seq data of TCGA PDAC datasets. **C** Gene set enrichment analysis (GSEA) of TCGA datasets showed that higher RPN6 expression was significantly associated with cell cycle, double-strand break repair, and homologous PDAC. **D** QRT-PCR and Western blot analysis of circ-MTHFD1L in the two gemcitabine-resistant cell lines compared with their parental cells. **E-F** PANC-1-GR cells with stable RPN6 overexpression or knockdown were constructed. **G-J** The proliferative ability of stably transfected PANC-1-GR or BxPC-3-GR cells dealing with gemcitabine was investigated via colony formation assays (**G**, **H**) and CCK-8 assays (cell viability and IC50) (**I**, **J**). Representative colony formation images are shown (**G**), and the numbers of colonies were summarized (**H**). **K** Flow cytometry analysis of the cell cycle progression of stably transfected PANC-1-GR or BxPC-3-GR cells dealing with gemcitabine was performed. Representative images and quantification of the results are presented. **L** Western blot analysis was performed to assess the protein levels of RAD50, RAD51, BRCA1, BRCA2, PCNA, P53 and P21 after knocking down or overexpressing RPN6 in PANC-1-GR and BxPC-3-GR cells. GAPDH was used as a loading control. **M** Confocal microscopic analysis of γH2AX staining showed that RPN6 decreased the distribution of γ-H2AX foci. Representative images and quantification of the results are presented. **N** The comet assay confirmed that RPN6 was closely related to the DNA repair capacity. Representative images and quantification of the results are presented. Data are shown as mean ± SD. *P < 0.05; **P < 0.01; ***P < 0.001, between the indicated groups

To further clarify the downstream mechanism of circ-MTHFD1L maintaining gemcitabine resistance, we divided the pancreatic cancer patients in The Cancer Genome Atlas (TCGA) database into two groups with high RPN6 expression and low RPN6 expression, and then performed Gene set enrichment analysis (GSEA), the results show that the high expression of RPN6 is highly correlated with cell cycle, DNA double-strand break repair and homologous recombination (Fig. 6A-C). Then we found that RPN6 was significantly upregulated in gemcitabine-resistant cell lines through qRT-PCR and western blotting (Fig. 6D). To study the effect of RPN6 on gemcitabine resistance, We stably knocked down RPN6 in PANC-1-GR and BxPC-3-GR through lentivirus-mediated transduction (Fig. 6E and Fig. S4A), among which sh-RPN6–2 had the most significant knockdown effect. Therefore, the sh-RPN6–2 cell line was used in our subsequent experiments. In addition, we also stably overexpressed RPN6 in PANC-1-GR and BxPC-3-GR through lentivirus-mediated transduction (Fig. 6F and Fig. S4B). Colony formation assays showed that the number of colonies was significantly reduced after RPN6 knockdown under gemcitabine loading, whereas the number of colonies was significantly increased after RPN6 overexpression (Fig. 6G, H). Cell viability testing produced similar results (Fig. 6I). Next, we further clarified the effect of RPN6 on gemcitabine resistance by testing the IC50 value. After RPN6 knockdown, PANC-1-GR and BxPC-3-GR cells were significantly less resistant to gemcitabine, while RPN6 overexpression significantly increased. (Fig. 6J). Flow cytometry showed that RPN6 knockdown significantly reduced the proportion of cells in the S phase under gemcitabine loading, while RPN6 overexpression induced the opposite cell cycle changes (Fig. 6K). To further prove that RPN6 can give cells a strong repairability to fight against DNA damage, we stimulated the cells with gemcitabine for 12 h or 24 h to cause the accumulation of DNA damage. We then replaced the gemcitabine-containing medium with a fresh medium to allow the cells to recover from damage within 24 h. Cellular DNA damage and repair levels were assessed by γH2AX (DNA damage marker) immunofluorescence assay and comet assay. The immunofluorescence results showed that the gemcitabine resistant cell lines with knockdown of RPN6 enhanced γH2AX induction under gemcitabine loading, indicating more DNA damage accumulation, and after gemcitabine elution, the fluorescence intensity of γH2AX was still higher than that of the control group, indicating that the DNA Damage repair ability is weak. The opposite result was shown in the gemcitabine-resistant cell lines overexpressing RPN6 (Fig. 6M). Comet experiments further confirmed this phenomenon. RPN6 knockdown in gemcitabine resistant cell lines caused more tail moments, indicating that the level of DNA damage increased, and after gemcitabine eluted, compared with the control group, more tail moment indicates weak DNA damage repairability. After overexpression of RPN6 in the drug-resistant cell line, the tail moment showed the opposite trend (Fig. 6N). In addition, western blot analysis showed that the downregulation of RPN6 reduced the expression of Rad50, Rad51, BRCA1, BRCA2 and PCNA and increased the expression of P53 and P21, while the upregulation of RPN6 had the opposite effect (Fig. 6L). The above results indicate that RPN6 can enhance the gemcitabine resistance of cells by affecting cell cycle progression and improving DNA damage repairability.

### Circ-MTHFD1L maintains gemcitabine resistance through miR-615-3p/RPN6 axis

**Fig. 7:**
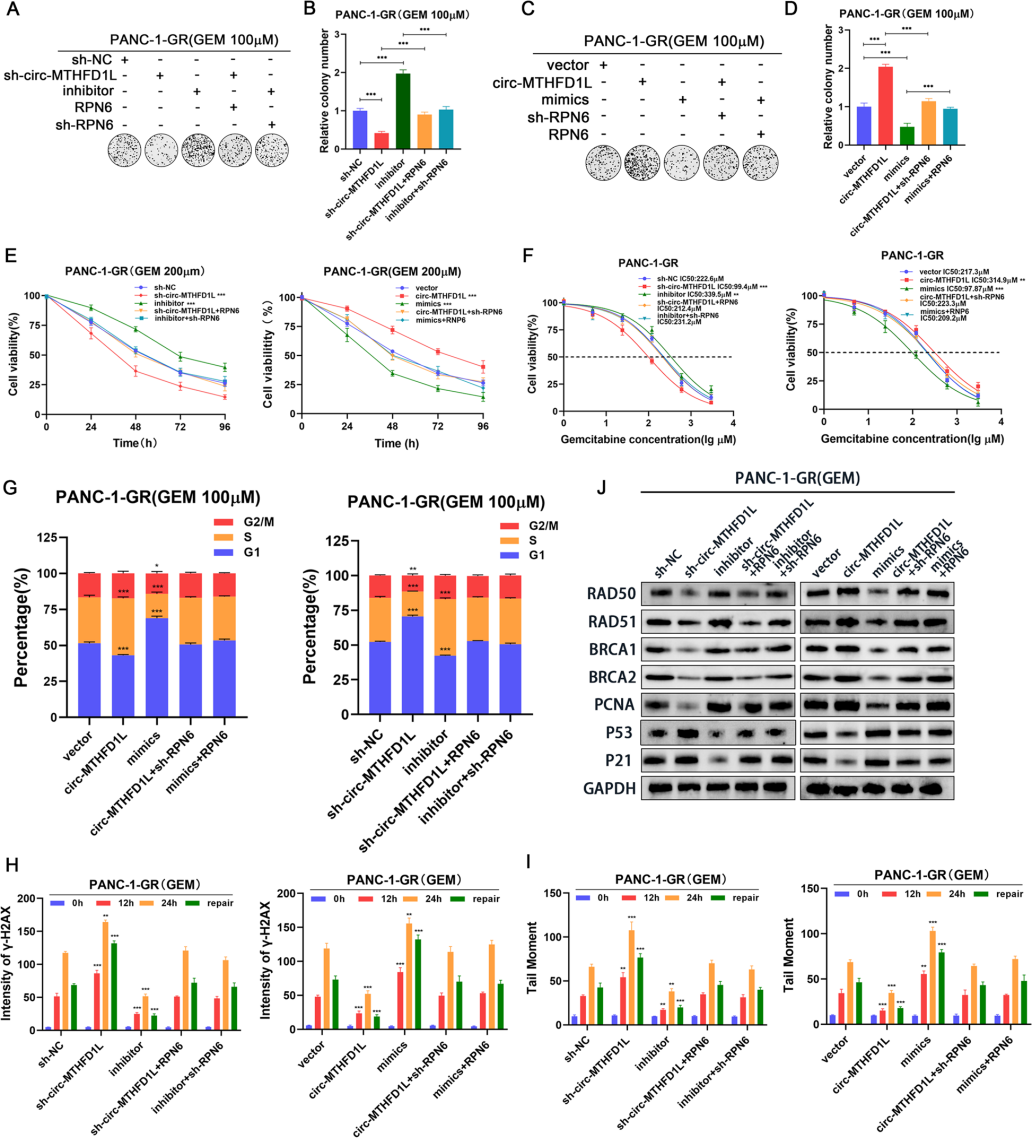
Circ-MTHFD1L maintains gemcitabine resistance through miR-615-3p/RPN6 axis. **A-F** The proliferative ability of the indicated cells dealing with gemcitabine was investigated via colony formation assays (**A-F**) and CCK-8 assays (cell viability and IC50) (**E-F**). Representative colony formation images are shown (**A**, **C**), and the numbers of colonies were summarized (**B**, **D**). **G** Flow cytometry analysis of the cell cycle progression of the indicated cells dealing with gemcitabine was performed. **I** Confocal microscopic analysis of γH2AX staining and the comet assay in the indicated cells was evaluated. The quantification of the results is presented. Data are shown as mean ± SD. *P < 0.05; **P < 0.01; ***P < 0.001, between the indicated groups. **J** The RAD50, RAD51, BRCA1, BRCA2, PCNA, P53 and P21 protein levels in PANC-1-GR and BxPC-3-GR cells from different groups were determined by western blot analysis. GAPDH was used as a loading control

To further demonstrate that circ-MTHFD1L maintains gemcitabine resistance by regulating the miR-615-3p-RPN6 axis, we constructed cells for rescue experiments and verified the transfection efficiency by qRT-PCR (Fig. S5A-D). Then We conducted a series of rescue experiments. The results of colony formation assays, cell viability test and IC50 value showed that RPN6 overexpression significantly restored the inhibitory effects induced by circ-MTHFD1L knockdown and miR-615-3p overexpression, while RPN6 knockdown was the opposite (Fig. 7A-F). Flow cytometry analysis, γH2AX immunofluorescence assay and comet assay showed that RPN6 overexpression significantly restored cell cycle arrest and weaker DNA damage repair levels caused by circ-MTHFD1L knockdown and miR-615-3p overexpression, while knockdown of RPN6 leads to the opposite result (Fig. 7G-I). In addition, western blot analysis confirmed that overexpression of RPN6 significantly restored the changes of cell cycle and homologous recombination repair-related proteins caused by circ-MTHFD1L silencing and miR-615-3p overexpression, while knockdown of RPN6 could reverse the changes of corresponding protein caused by circ-MTHFD1L overexpression and miR-615-3p inhibition (Fig. 7J). In summary, these results together prove that circ-MTHFD1L promotes cell cycle progression and strengthens DNA damage repair through the regulation of the miR-615-3p/RPN6 axis, thereby maintaining gemcitabine resistance.

### Pancreatic cancer CDXs with circ-MTHFD1L silencing is more sensitive to olaparib combined with gemcitabine therapy

**Fig. 8:**
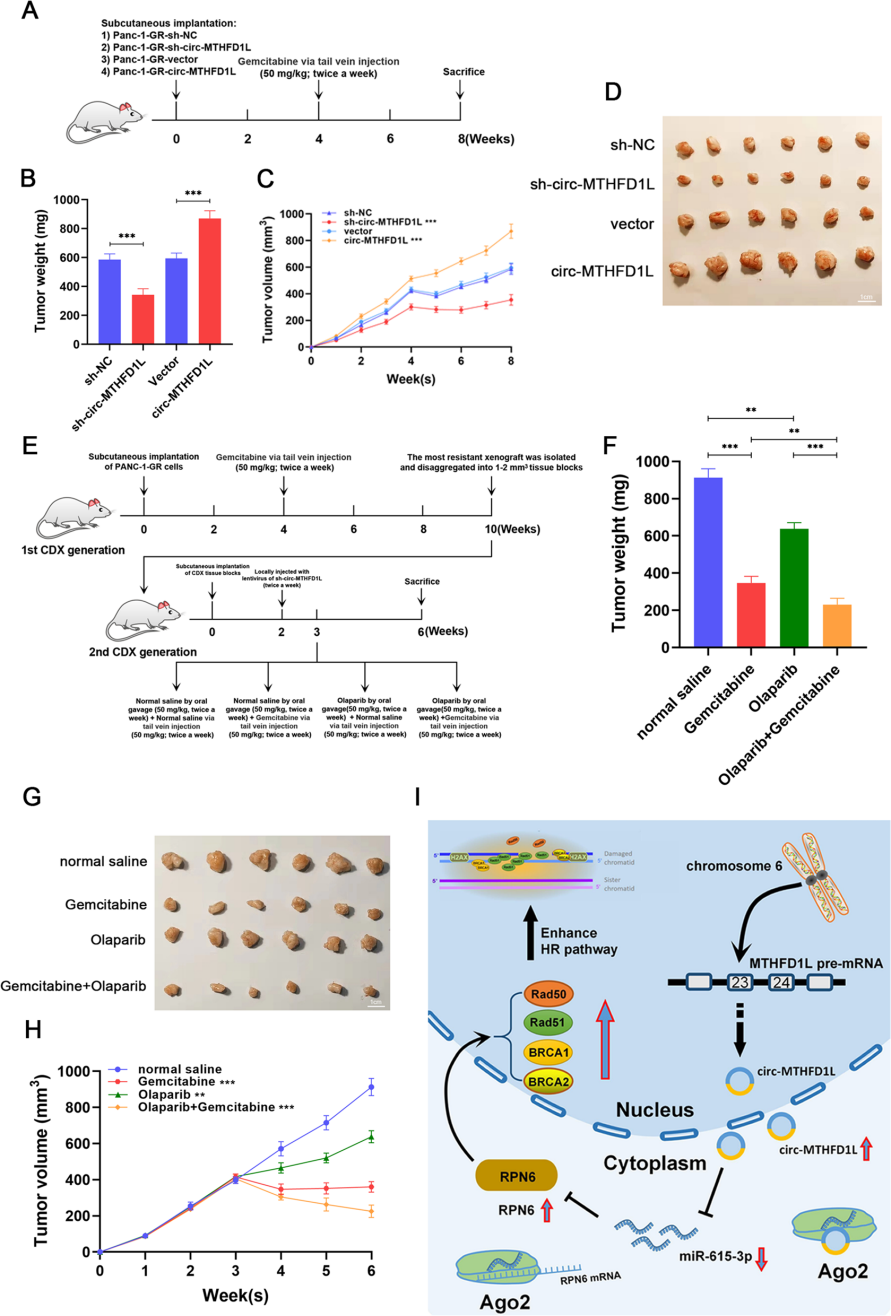
Pancreatic cancer CDXs with circ-MTHFD1L silencing is more sensitive to olaparib combined with gemcitabine therapy. **A-D** BALB/c nude mice (4–6 week-old males) were subcutaneously injected with stably transfected PANC-1-GR cells. Four weeks after implantation, the mice were treated with Gemcitabine (50 mg/kg/twice a week) by tail vein injection. Mice were sacrificed on week 8, and xenografts were isolated and measured. **E-H** BALB/c nude mice (4–6 week-old males) were subcutaneously injected with PANC-1-GR cells and treated with gemcitabine (50 mg/kg/twice a week) tail vein injection starting on week 4. Six weeks later, the most resistant xenograft was disaggregated and implanted subcutaneously into BALB/c nude mice (4–6 week-old males) as the second GR-CDX. Two weeks after implantation, the second GR-CDX generation mice were treated with sh-circ-MTHFD1L lentivirus (twice a week) and randomized into four groups on week 3. The four groups of mice were treated as follows: gemcitabine alone, olaparib alone, gemcitabine combined with olaparib, and negative control. Mice were sacrificed on week 6, and xenografts were isolated and measured. Data are shown as mean ± SD. *P < 0.05; **P < 0.01; ***P < 0.001, between the indicated groups. **I** Schematic illustration of the circ-MTHFD1L/miR-615-3p/RPN6 axis in PDAC cells

To explore the potential role of circ-MTHFD1L in vivo, we constructed a subcutaneous cdx model (Fig. 8A). We found that subcutaneous transplantation of circ-MTHFD1L silenced PANC-1-GR cells was significantly more sensitive to gemcitabine treatment than the control group, while overexpression of circ-MTHFD1L showed the opposite effect (Fig. 8B-D). To further discover potential clinical applications, we speculate that PARP inhibitors increase gemcitabine sensitivity after circ-MTHFD1L silence by further inhibiting DNA damage repair in drug-resistant cells. After constructing two generations of subcutaneous gemcitabine-resistant CDX models (Fig. 8E), we found that the treatment of olaparib has a significant effect. Moreover, olaparib combined with gemcitabine treatment can further improve the therapeutic effect after circ-MTHFD1L silence. (Fig. 8F-H). These results indicate that circ-MTHFD1L maintains gemcitabine resistance in vivo and PDAC is more sensitive to olaparib combined with gemcitabine therapy after circ-MTHFD1L silencing.

## Discussion

In recent years, pancreatic cancer morbidity and lethality have increased, but effective treatment has not made significant progress. Especially for the treatment of advanced pancreatic cancer, it has become an important topic in tumor research. Although with the popularization of genetic testing technology, the advent of targeted drugs, and breakthroughs in immunotherapy-related technologies, precision medicine has brought dawn to the treatment of tumors. However, these drugs still have significant limitations for most patients [5–7]. Chemotherapy as a treatment for advanced tumors still has an unshakable position. However, pancreatic cancer cells that were initially sensitive to chemotherapy will eventually lead to tumor progression due to drug resistance. Exploring the mechanisms related to chemotherapy resistance is still the focus and difficulty that the discipline needs to face [30]. Thus, there is an urgent need to find targets and markers related to chemotherapy resistance in pancreatic cancer. The main mechanism for chemotherapy to damage tumor cells is DNA damage, and a natural DNA repair mechanism in tumor cells can quickly repair DNA damage caused by chemotherapy and lead to chemotherapy resistance [31]. Therefore, the focus of solving the chemotherapy resistance of pancreatic cancer is to explore genes related to DNA damage and repair. With the continuous advancement of high-throughput sequencing technology and the continuous development of bioinformatics technology, people’s understanding of circRNAs has also been deepened, and more and more circRNAs have been explored by scientists [32]**.** Numerous studies have shown that circRNAs play an indispensable role in maintaining health [33]. The research on the new circRNAs aspect of pancreatic cancer has important clinical practice significance, especially the chemotherapy resistance of pancreatic cancer. Through in-depth exploration, we discovered for the first time the key role of the circ-MTHFD1L/miR-615-3p/RPN6 axis in the process of chemotherapy resistance in pancreatic cancer.

This study constructed gemcitabine-resistant pancreatic cancer cell lines (PANC-1-GR, BxPC-3-GR) by in vitro low-concentration gradient escalation combined with high-dose intermittent shock. IC50 has increased by nearly 200 times compared with wild-type pancreatic cancer cell lines. Combining the GEO database and the sequencing data of drug-resistant cell lines in our center, we found a new circRNA: hsa_circ_0078297 (circ-MTHFD1L). Many ncRNAs have low expression levels in cancer and normal cells [34]. However, through the analysis of the qRT-PCR results of clinical samples, we confirmed that circ-MTHFD1L is a circRNA that is significantly upregulated and stably expressed in PDAC tissues. And the high expression level of circ-MTHFD1L is significantly correlated with chemotherapy resistance and poor prognosis of PDAC patients. By consulting the databases (Circbase and UCSC), we found that circ-MTHFD1L was generated by the back splicing of the 23rd and 24th exons of the human MTHFD1L gene on chromosome 6q25.1. The circular structure and better stability of circ-MTHFD1L were verified by a series of experiments. Circ-MTHFD1L can be stably expressed in various pancreatic cancer cell lines and localized in the cytoplasm. In addition, the resistance-related functional assays showed that circ-MTHFD1L as an oncogene enhances gemcitabine resistance in pancreatic cancer cells. By constructing a CDX mouse model to simulate the resistance of patients with pancreatic cancer to gemcitabine, we found that CDX mice with high expression of circ-MTHFD1L are significantly resistant to gemcitabine treatment. It suggests that circ-MTHFD1L may be involved in PDAC gemcitabine chemotherapy resistance.

CircRNA often acts as a miRNA sponge to promote the occurrence and development of tumors by antagonizing the expression and function of miRNA [29, 34]. We predicted the potential downstream targets of circ-MTHFD1L through bioinformatics tools (ENCORI, CircInteractome, Circbank, CircAtlas) and initially identified miR-516b-5p and miR-615-3p as candidate targets.

We found that only miR-615-3p is significantly enriched in the two gemcitabine-resistant cell lines through RNA pull-down experiments. Through the dual-luciferase report assay and RIP experiments, we confirmed the interaction of miR-615-3p and circ-MTHFD1L. In addition, the Fish experiment proved that they are co-localized in the cytoplasm. Combined analysis of the GEO database and clinical samples confirmed that miR-615-3p was significantly reduced in PDAC. The statistical analysis of the results of qRT-PCR of clinical samples also showed a negative correlation between the expression of circ-MTHFD1L and miR-615-3p in PDAC tissues. The abnormal expression of miR-615-3p has been confirmed to be related to the occurrence and development of gastric cancer, breast cancer, and non-small cell lung cancer [15–17]. However, there are no reports about it and pancreatic cancer globally. Cell function experiments showed that miR-615-3p significantly affected the drug resistance of pancreatic cancer cells. In vitro rescue experiments confirmed that miR-615-3p could reverse the resistance of circ-MTHFD1L.

RPN6, also known as PSMD11, is described as 26S proteasome non-ATPase regulatory subunit 11. It has been reported that its increased expression is associated with DNA replication pressure [35] and ubiquitination [36]. However, it is rarely reported in tumors, and reports related to drug resistance have not been published. The exploration of RPN6 is of great significance for perfecting tumor progression-related mechanisms. In this study, we found that RPN6 is a potential downstream target gene of miR-615-3p through the prediction of multiple bioinformatics tools (ENCORI, CircInteractome, Circbank, CircAtlas). The Dual-luciferase reporter assay confirmed that RPN6 is the downstream target of miR-615-3p. The results of qRT-PCR and western blot showed that the expression of Circ-MTHFD1L could affect the expression of RNP6, while the change of miR-615-3p could reverse this phenomenon, suggesting that there was an obvious regulatory relationship between them. In RPN6 overexpressing and knockdown cells, the correlation between RPN6 and HR pathways was determined by γH2AX immunofluorescence assay, comet assay and, and HR pathway-related molecular markers detection. Rescue experiments also demonstrated that the altered gemcitabine resistance in pancreatic cancer caused by RPN6 gain and loss could be reversed by circ-MTHFD1L and miR-615-3p. Based on the bioinformatics analysis results of the TCGA database data combined with previous reports [37], we speculate that RNP6 may mediate the ubiquitination of HR pathway-related proteins to mediate the chemotherapy resistance [38] of pancreatic cancer gemcitabine and affect its cell cycle progression, which requires our further experiments to verify. Our study demonstrates that circ-MTHFD1L can reduce the inhibition of miR-615-3p on RPN6 by acting as a miR-615-3p sponge, thereby enhancing the resistance of gemcitabine to pancreatic cancer.

Cancer drug resistance is a multi-factor and dynamic evolutionary phenomenon that involves pre-existing or treatment-obtained mutations and non-genetic and epigenetic mechanisms that reduce the response to treatment. Tumor heterogeneity and TME are also related to drug resistance [30]. Chemoresistance of pancreatic cancer cells is an important reason that hinders the efficacy of gemcitabine chemotherapy [10]. DNA damage repair plays a very critical role in it. As one of the two ways of DNA repair, single-strand repair often plays a greater role in minor and early damages. DNA single-strand breaks are repaired through mismatch, base excision, and nucleotide excision [31, 39]. Poly ADP-ribose polymerase (PARP) is an enzyme closely related to DNA single-strand damage repair. PARP inhibitors are well known as targeted drugs for patients with BRCA mutations [40]. In our research, we found that silencing circ-MTHFD1L combined with olaparib enhances the therapeutic effect of gemcitabine on pancreatic cancer. We speculate that this may be related to the phenomenon of “BRCAness”.(BRCAness revisited.) Silencing circ-MTHFD1L caused a decrease in BRCA1/2 expression or other unknown changes in BRCAness-associated proteins, undoubtedly pointing out a new direction for circ-MTHFD1L as a therapeutic target for pancreatic cancer. Many previous studies have identified many targets and markers of drug resistance in pancreatic cancer [41–43], but in our research, through multiple verifications, combined with data including bioinformatics, cell experiments, animal experiments, and clinical sample data, we have obtained more reliable results than before, proving that the new circ-MTHFD1L plays an extremely important role in the chemotherapy resistance process of pancreatic cancer through the miR-615-3p/RPN6 axis. Of course, our experiment still has certain limitations. First, circRNAs are not only working as the miRNA sponge. In addition, there are other mechanisms of action that we need to explore, such as variable shearing, translation into protein [44–46]. Our research found that changes in RPN6 can indeed cause changes in pancreatic cancer drug resistance and changes in the expression of HR pathway-related proteins. However, how RPN6 causes changes in the HR pathway and the specific mechanism of circ-MTHFD1 causing the “BRCAness” phenomenon requires further in-depth research, and more in-depth exploration in the future will make this research more clinically diagnostic and therapeutic; Third, the clinical sample research involved in this research is limited to the cases of the center, which may be lacking in representativeness, but I believe that with the increase in scientific research investment and attention and the construction of a community with a shared future for humankind, we will have more opportunities to cooperate with other research centers, including China and the West, to obtain more clinical data and improve the applicability of this research in the future.

## Conclusion

In conclusion, circ-MTHFD1L competitively binds to miR-615-3p, attenuates the inhibitory effect of miR-615-3p on RPN6, and promotes gemcitabine chemoresistance in PDAC cells. As expected, knockdown of circ-MTHFD1L makes the interaction of olaparib and gemcitabine possible. Our findings expand the mechanisms of PDAC chemoresistance and provide new markers for diagnosing PDAC chemoresistance.

## Supplementary Information

**Additional file 1: Fig. S1. A** quantitative RT-PCR (qRT-PCR) analysis of MTHFD1L mRNA in the two gemcitabine-resistant cell lines compared with their parental cells. **B** Overall survival of PDAC patients (N = 178) from TCGA project with high or low MTHFD1L expression levels.**C**-**D** The overexpression and knockdown efficiency of circ-MTHFD1L were confirmed by qRT-PCR analysis of circ-MTHFD1L and MTHFD1L mRNA in BxPC-3-GR. Data are shown as mean ± SD. *P < 0.05; **P < 0.01; ***P < 0.001, between the indicated groups.

**Additional file 2: Fig. S2. A** List of circ-MTHFD1L binding proteins predicted by the CSCD database.**B** Schematic diagram of circ-MTHFD1L binding proteins predicted by CircAtlas database

**Additional file 3: Fig. S3. A**-**D** The transfection efficiency of circ-MTHFD1L and miR-615-3p in the indicated group was verified by qRT-PCR. Data are shown as mean ± SD. *P < 0.05; **P < 0.01; ***P < 0.001, between the indicated groups.

**Additional file 4: Fig. S4. A**-**B** PANC-1-GR cells with stable RPN6 overexpression or knockdown were constructed. Data are shown as mean ± SD. *P < 0.05; **P < 0.01; ***P < 0.001, between the indicated groups.

**Additional file 5: Fig. S5. A**-**D** The transfection efficiency of circ-MTHFD1L, miR-615-3p and RPN6 in the indicated group was verified by qRT-PCR. Data are shown as mean ± SD. *P < 0.05; **P < 0.01; ***P < 0.001, between the indicated groups.

**Additional file 6: Table S1.** The sequences of primers and oligonucleotides used in this study

**Additional file 7: Table S2.** Correlation of circ-MTHFD1L expression with clinicopathologic features of PDAC patients

**Additional file 8: Table S3.** Correlation of PFS with clinicopathologic features of PDAC patients.

**Additional file 9: Table S4.** MiRNAs predicted by ENCORI, CircInteractome, Circbank and CircAtlas databases that may bind to circ-MTHFD1L and the intersection of the four databases.

**Additional file 10: Table S5.** Downstream target genes of miR-615-3p predicted by ENCORI, miRDB, mirDIP, miRTarBase and TargetScan databases and the intersection of five databases.

## Data Availability

All data in our study are available upon request.

## Abbreviations

PDAC: Pancreatic ductal adenocarcinoma
GEO: Gene Expression Omnibus
FISH: Fluorescence in situ hybridization
RIP: RNA immunoprecipitation
HPDE: Human pancreatic ductal epithelial
PANC-1-GR: Gemcitabine-resistant Panc-1
BxPC-3-GR: Gemcitabine-resistant Bxpc-3
DEGs: Differentially expressed genes
ECL: Enhanced chemiluminescence
UTR: Untranslated region
CDX: Cell line-derived xenograft
LSD: Least significant difference
OS: Overall survival
PFS: Progression-free survival
DFS: Disease-free survival

## References

[1] Siegel R, Miller K, Jemal A (2019). Cancer statistics, 2019. CA Cancer J Clin.

[2] Sung H, Ferlay J, Siegel R, Laversanne M, Soerjomataram I, Jemal A, et al. Global Cancer Statistics 2020: GLOBOCAN estimates of incidence and mortality worldwide for 36 cancers in 185 countries. CA Cancer J Clin. 2021;(71):209–49. 10.3322/caac.21660 33538338

[3] Mizrahi J, Surana R, Valle J, Shroff R (2020). Pancreatic cancer. Lancet (London, England).

[4] Kim V, Ahuja N: Early detection of pancreatic cancer Chinese journal of cancer research = Chung-kuo yen cheng yen chiu 2015, 27:321–331. 10.3978/j.issn.1000-9604.2015.07.03 PMC4560741 26361402

[5] Henriksen A, Dyhl-Polk A, Chen I, Nielsen D (2019). Checkpoint inhibitors in pancreatic cancer. Cancer Treat Rev.

[6] Akce M, Zaidi M, Waller E, El-Rayes B, Lesinski G (2018). The potential of CAR T cell therapy in pancreatic Cancer. Front Immunol.

[7] Foley K, Kim V, Jaffee E, Zheng L (2016). Current progress in immunotherapy for pancreatic cancer. Cancer Lett.

[8] Chen S, Yang C, Wang Z, Hu J, Pan J, Liao C, Zhang J, Chen J, Huang Y, Huang L (2021). CLK1/SRSF5 pathway induces aberrant exon skipping of METTL14 and cyclin L2 and promotes growth and metastasis of pancreatic cancer. J Hematol Oncol.

[9] Binenbaum Y, Na'ara S, Gil Z (2015). Gemcitabine resistance in pancreatic ductal adenocarcinoma. Drug Resist Updat.

[10] De Dosso S, Siebenhüner A, Winder T, Meisel A, Fritsch R, Astaras C, Szturz P, Borner M (2021). Treatment landscape of metastatic pancreatic cancer. Cancer Treat Rev.

[11] Chen I, Chen C, Chuang T (2015). Biogenesis, identification, and function of exonic circular RNAs. Wiley Interdiscip Rev RNA.

[12] Memczak S, Jens M, Elefsinioti A, Torti F, Krueger J, Rybak A, Maier L, Mackowiak S, Gregersen L, Munschauer M (2013). Circular RNAs are a large class of animal RNAs with regulatory potency. Nature.

[13] Rybak-Wolf A, Stottmeister C, Glažar P, Jens M, Pino N, Giusti S, Hanan M, Behm M, Bartok O, Ashwal-Fluss R (2015). Circular RNAs in the mammalian brain are highly abundant, conserved, and dynamically expressed. Mol Cell.

[14] Chen W, Quan Y, Fan S, Wang H, Liang J, Huang L, Chen L, Liu Q, He P, Ye Y (2020). Exosome-transmitted circular RNA hsa_circ_0051443 suppresses hepatocellular carcinoma progression. Cancer Lett.

[15] Zhang N, Nan A, Chen L, Li X, Jia Y, Qiu M, Dai X, Zhou H, Zhu J, Zhang H, Jiang Y (2020). Circular RNA circSATB2 promotes progression of non-small cell lung cancer cells. Mol Cancer.

[16] Gong L, Chen J, Dong M, Xiao Z, Feng Z, Pan Y, Zhang Y, Du Y, Zhang J, Bi Y (2020). Epstein-Barr virus-derived circular RNA LMP2A induces stemness in EBV-associated gastric cancer. EMBO Rep.

[17] Xu Y, Zhang S, Liao X, Li M, Chen S, Li X, Wu X, Yang M, Tang M, Hu Y (2021). Circular RNA circIKBKB promotes breast cancer bone metastasis through sustaining NF-κB/bone remodeling factors signaling. Mol Cancer.

[18] Vo J, Cieslik M, Zhang Y, Shukla S, Xiao L, Zhang Y, Wu Y, Dhanasekaran S, Engelke C, Cao X (2019). The landscape of circular RNA in Cancer. Cell.

[19] Reese M, Dhayat S (2021). Small extracellular vesicle non-coding RNAs in pancreatic cancer: molecular mechanisms and clinical implications. J Hematol Oncol.

[20] Chen S, Chen C, Hu Y, Song G, Shen X (2021). The diverse roles of circular RNAs in pancreatic cancer. Pharmacol Ther.

[21] Macchini M, Centonze F, Peretti U, Orsi G, Militello A, Valente M, Cascinu S, Reni M (2021). Treatment opportunities and future perspectives for pancreatic cancer patients with germline BRCA1-2 pathogenic variants. Cancer Treat Rev.

[22] Lord C, Ashworth A (2016). BRCAness revisited. *Nature reviews*. Cancer.

[23] Byrum A, Vindigni A, Mosammaparast N (2019). Defining and modulating 'BRCAness'. Trends Cell Biol.

[24] Gyori B, Venkatachalam G, Thiagarajan P, Hsu D, Clement M (2014). OpenComet: an automated tool for comet assay image analysis. Redox Biol.

[25] Casper J, Zweig A, Villarreal C, Tyner C, Speir M, Rosenbloom K, Raney B, Lee C, Lee B, Karolchik D (2018). The UCSC genome browser database: 2018 update. Nucleic Acids Res.

[26] Glažar P, Papavasileiou P, Rajewsky N (2014). circBase: a database for circular RNAs. RNA (New York, NY).

[27] Hui Y, Reitz J (1997). Gemcitabine: a cytidine analogue active against solid tumors American journal of health-system pharmacy. Am J Health Syst Pharm.

[28] Hamed S, Straubinger R, Jusko W (2013). Pharmacodynamic modeling of cell cycle and apoptotic effects of gemcitabine on pancreatic adenocarcinoma cells. Cancer Chemother Pharmacol.

[29] Zhong Y, Du Y, Yang X, Mo Y, Fan C, Xiong F, Ren D, Ye X, Li C, Wang Y (2018). Circular RNAs function as ceRNAs to regulate and control human cancer progression. Mol Cancer.

[30] Zeng S, Pöttler M, Lan B, Grützmann R, Pilarsky C, Yang H: Chemoresistance in Pancreatic Cancer. International journal of molecular sciences 2019, 20. 10.3390/ijms20184504 PMC6770382 31514451

[31] Roos W, Thomas A, Kaina B (2016). DNA damage and the balance between survival and death in cancer biology. Nat Rev Cancer.

[32] Wang Y, An Y, Li B, Lu J, Guo J (2019). Research progress on circularRNAs in pancreatic cancer: emerging but promising. Cancer Biol Therapy.

[33] He L, Man C, Xiang S, Yao L, Wang X, Fan Y (2021). Circular RNAs' cap-independent translation protein and its roles in carcinomas. Mol Cancer.

[34] Goodall G, Wickramasinghe V (2021). RNA in cancer. *Nature reviews*. Cancer.

[35] Tkach J, Yimit A, Lee A, Riffle M, Costanzo M, Jaschob D, Hendry J, Ou J, Moffat J, Boone C (2012). Dissecting DNA damage response pathways by analysing protein localization and abundance changes during DNA replication stress. Nat Cell Biol.

[36] Ding Z, Xu C, Sahu I, Wang Y, Fu Z, Huang M, Wong C, Glickman M, Cong Y (2019). Structural snapshots of 26S proteasome reveal Tetraubiquitin-induced conformations. Mol Cell.

[37] Aquila L, Atanassov B. Regulation of histone ubiquitination in response to DNA double Strand breaks. Cells. 2020;9. 10.3390/cells9071699 PMC7407225 32708614

[38] Gallo L, Ko J, Donoghue D (2017). The importance of regulatory ubiquitination in cancer and metastasis. Cell cycle (Georgetown, Tex).

[39] Turgeon M, Perry N, Poulogiannis G (2018). DNA damage, repair, and Cancer metabolism. Front Oncol.

[40] Genta S, Martorana F, Stathis A, Colombo I. Targeting the DNA damage response: PARP inhibitors and new perspectives in the landscape of cancer treatment. Crit Rev Oncol Hematol. 2021;103539. 10.1016/j.critrevonc.2021.103539 34800653

[41] Yang J, Xu J, Zhang B, Tan Z, Meng Q, Hua J, et al. Ferroptosis: at the crossroad of gemcitabine resistance and tumorigenesis in pancreatic Cancer. Int J Mol Sci. 2021;22. 10.3390/ijms222010944 PMC8539929 34681603

[42] Rozengurt E. Eibl G: Crosstalk between KRAS, SRC and YAP signaling in pancreatic Cancer: interactions leading to aggressive disease and drug resistance. Cancers. 2021;13. 10.3390/cancers13205126 PMC8533944 34680275

[43] Jia Y, Gu D, Wan J, Yu B, Zhang X, Chiorean E, Wang Y, Xie J (2019). The role of GLI-SOX2 signaling axis for gemcitabine resistance in pancreatic cancer. Oncogene.

[44] Li X, Yang L, Chen L (2018). The biogenesis, functions, and challenges of circular RNAs. Mol Cell.

[45] Ho J, Di Tullio F, Schwarz M, Low D, Incarnato D, Gay F, et al. HNRNPM controls circRNA biogenesis and splicing fidelity to sustain cancer cell fitness. eLife. 2021;10. 10.7554/eLife.59654 PMC8346284 34075878

[46] Zhao W, Cui Y, Liu L, Qi X, Liu J, Ma S, Hu X, Zhang Z, Wang Y, Li H (2020). Splicing factor derived circular RNA circUHRF1 accelerates oral squamous cell carcinoma tumorigenesis via feedback loop. Cell Death Differ.

