# Supplementary figures and images for: Circular RNA circ-MTHFD1L induces HR repair to promote gemcitabine resistance via the miR-615-3p/RPN6 axis in pancreatic ductal adenocarcinoma
Source: J Exp Clin Cancer Res. 2022 Apr 23;41:153. doi: 10.1186/s13046-022-02343-z (PMC9034615; doi:10.1186/s13046-022-02343-z)

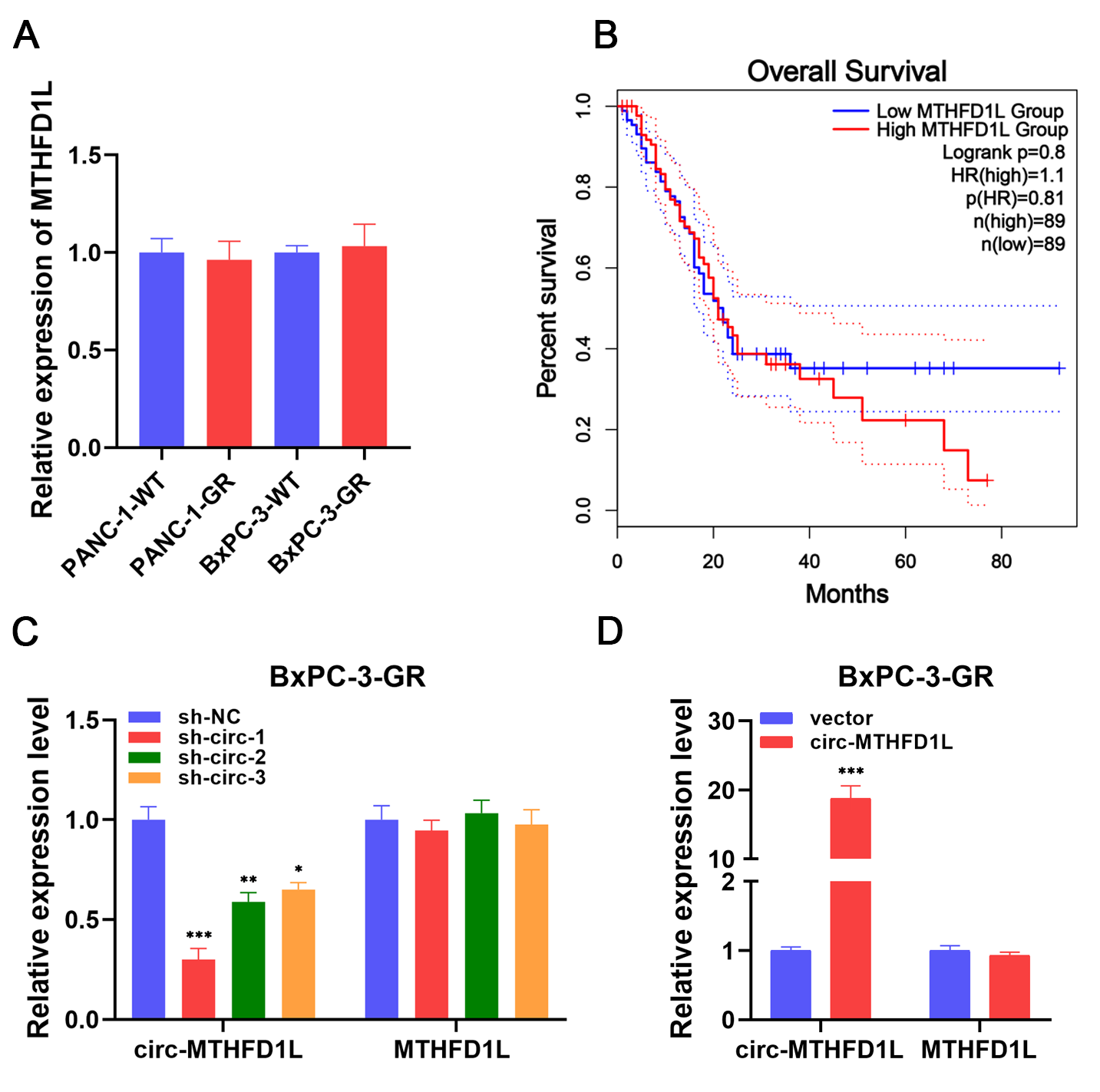

Supplement: Supplementary file 1 — Additional file 1: Fig. S1. A quantitative RT-PCR (qRT-PCR) analysis of MTHFD1L mRNA in the two gemcitabine-resistant cell lines compared with their parental cells. B Overall survival of PDAC patients (N = 178) from TCGA project with high or low MTHFD1L expression levels.C-D The overexpression and knockdown efficiency of circ-MTHFD1L were confirmed by qRT-PCR analysis of circ-MTHFD1L and MTHFD1L mRNA in BxPC-3-GR. Data are shown as mean ± SD. *P < 0.05; **P < 0.01; ***P < 0.001, between the indicated groups. [file 13046_2022_2343_MOESM1_ESM.tif]

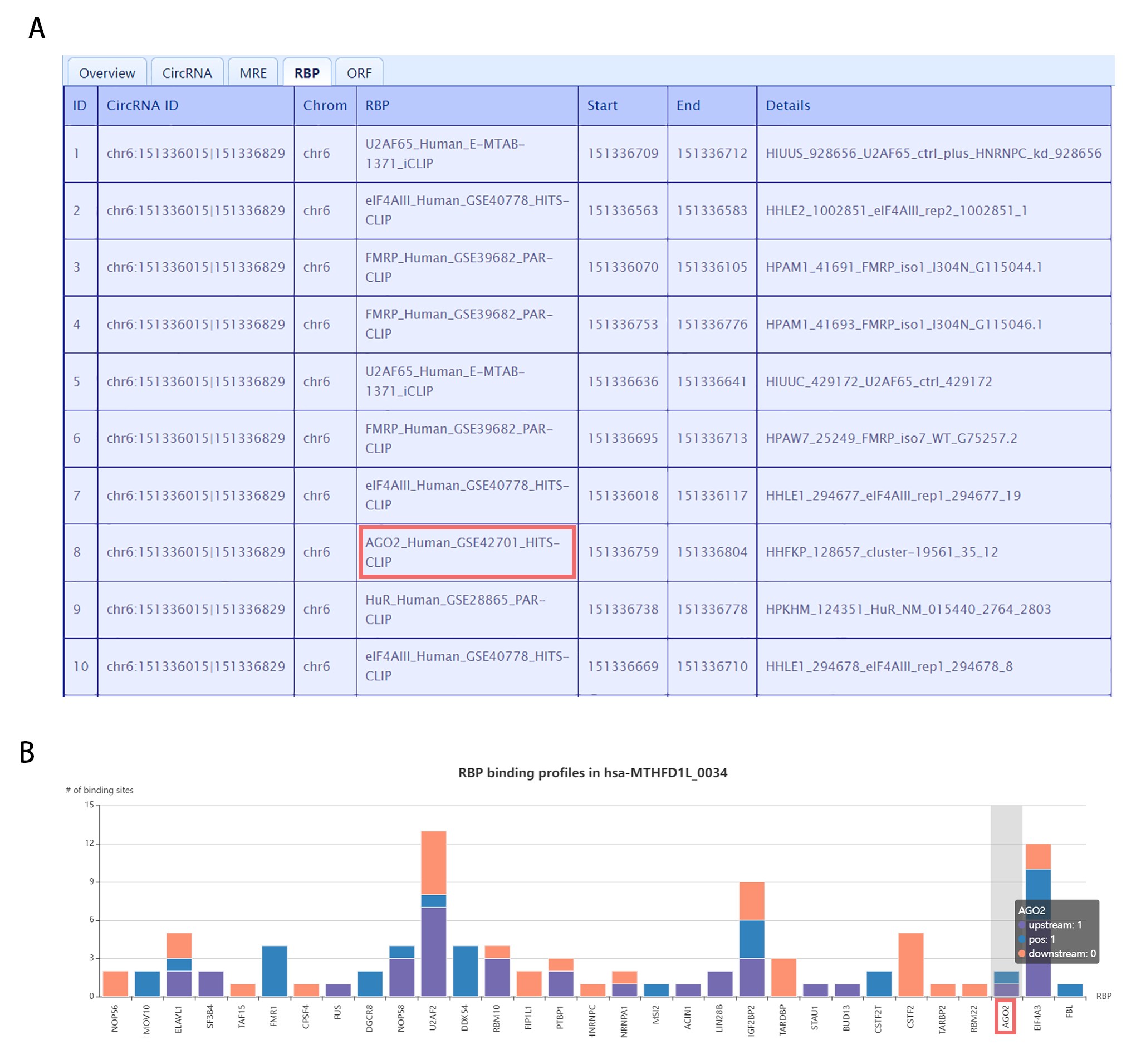

Supplement: Supplementary file 2 — Additional file 2: Fig. S2. A List of circ-MTHFD1L binding proteins predicted by the CSCD database.B Schematic diagram of circ-MTHFD1L binding proteins predicted by CircAtlas database [file 13046_2022_2343_MOESM2_ESM.tif]

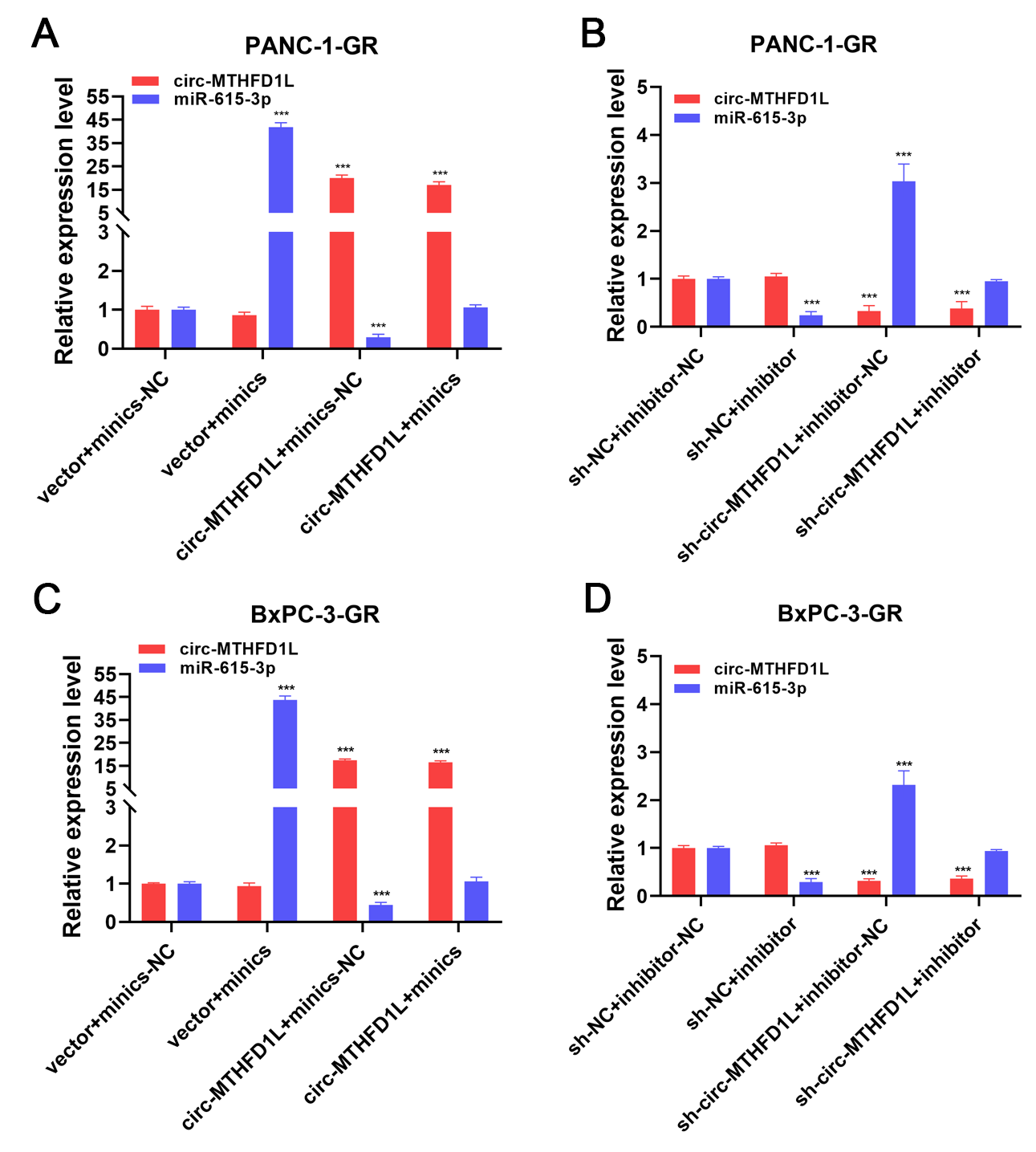

Supplement: Supplementary file 3 — Additional file 3: Fig. S3. A-D The transfection efficiency of circ-MTHFD1L and miR-615-3p in the indicated group was verified by qRT-PCR. Data are shown as mean ± SD. *P < 0.05; **P < 0.01; ***P < 0.001, between the indicated groups. [file 13046_2022_2343_MOESM3_ESM.tif]

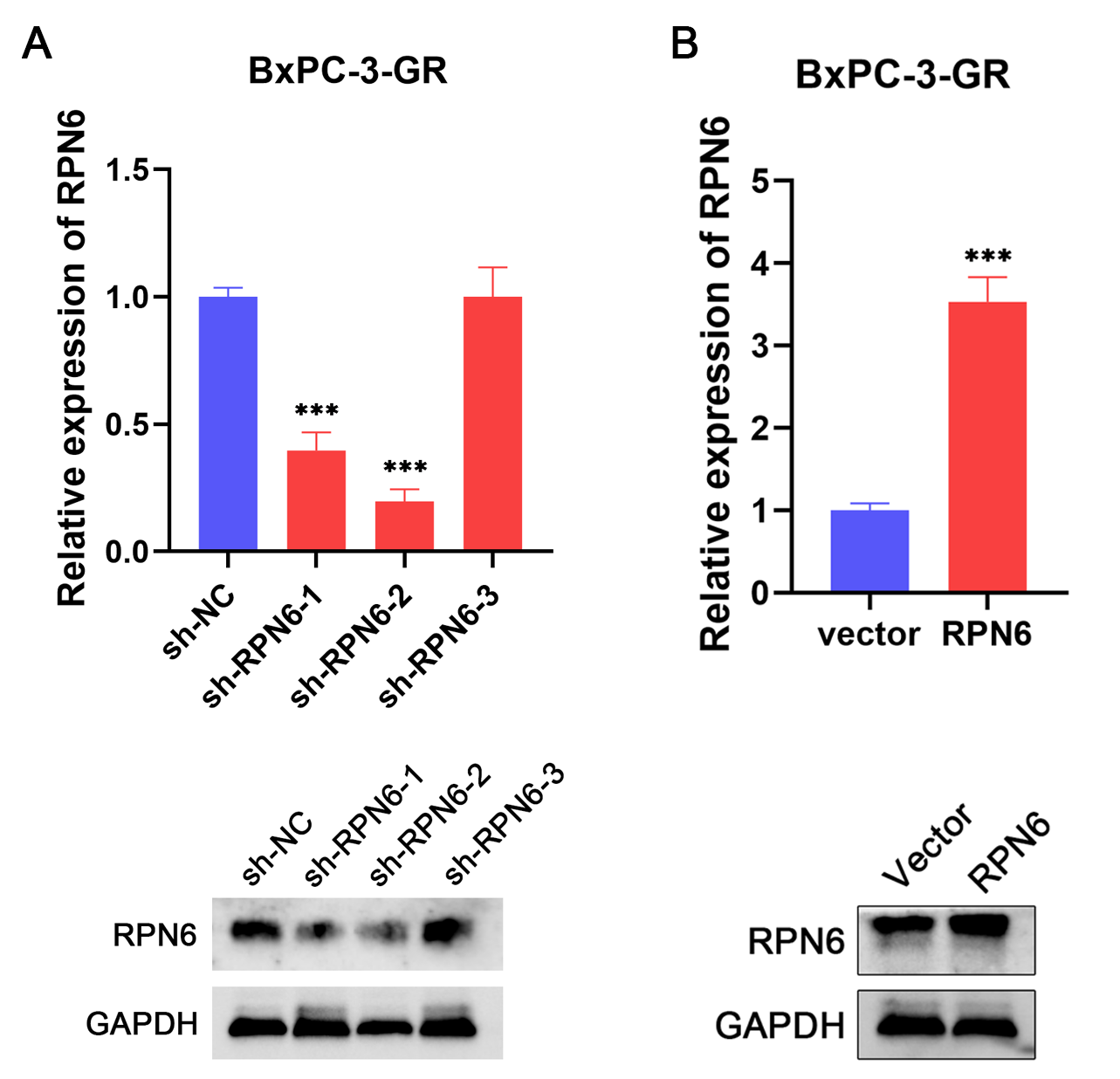

Supplement: Supplementary file 4 — Additional file 4: Fig. S4. A-B PANC-1-GR cells with stable RPN6 overexpression or knockdown were constructed. Data are shown as mean ± SD. *P < 0.05; **P < 0.01; ***P < 0.001, between the indicated groups. [file 13046_2022_2343_MOESM4_ESM.tif]

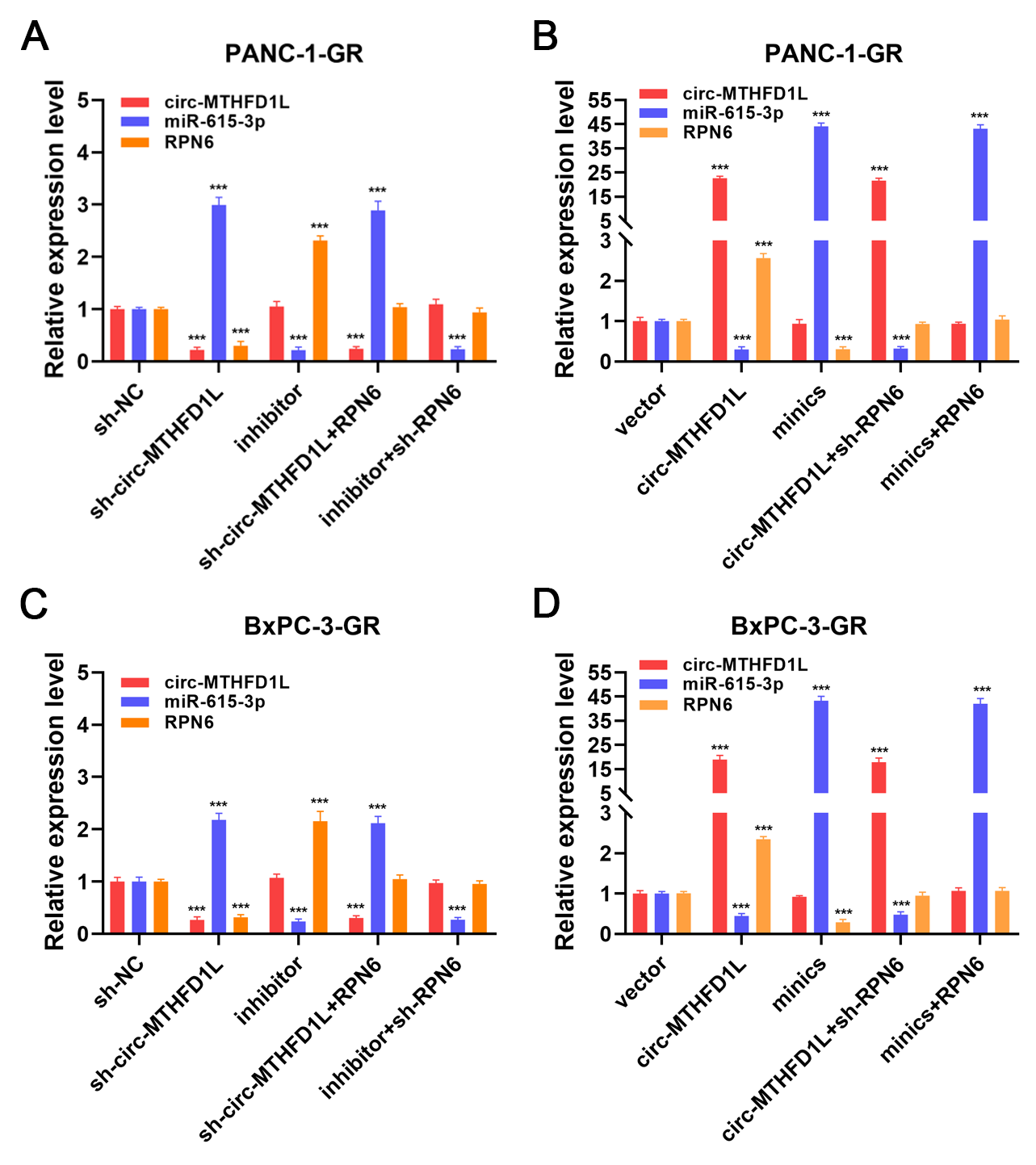

Supplement: Supplementary file 5 — Additional file 5: Fig. S5. A-D The transfection efficiency of circ-MTHFD1L, miR-615-3p and RPN6 in the indicated group was verified by qRT-PCR. Data are shown as mean ± SD. *P < 0.05; **P < 0.01; ***P < 0.001, between the indicated groups. [file 13046_2022_2343_MOESM5_ESM.tif]
