# Supplementary material for: Circular RNA circ-MTHFD1L induces HR repair to promote gemcitabine resistance via the miR-615-3p/RPN6 axis in pancreatic ductal adenocarcinoma
Source: J Exp Clin Cancer Res. 2022 Apr 23;41:153. doi: 10.1186/s13046-022-02343-z (PMC9034615; doi:10.1186/s13046-022-02343-z)
Supplement: Supplementary file 6 — Additional file 6: Table S1. The sequences of primers and oligonucleotides used in this study [file 13046_2022_2343_MOESM6_ESM.pdf]

Table S1. The sequences of primers and oligonucleotides used in this study

The sequences of primers for qRT-PCR	
Name	Oligo Sequence (5’ -3’)
circ-MTHFD1L forward	GCTATCACTGGTCCGTTGGT
circ-MTHFD1L reverse	CTGAATTTGCTTCTGGAGGTTACA
miR-516b-5p forward	CCGCGCATCTGGAGGTAAGAAG
miR-516b-5p reverse	AGTGCAGGGTCCGAGGTATT
miR-516b-5p stem-loop	GTCGTATCCAGTGCAGGGTCCGAGGTATTCGCACTGGATACGACAAAGTG
miR-615-3p forward	GTTCTCCGAGCCTGGGTCTC
miR-615-3p reverse	AGTGCAGGGTCCGAGGTATT
miR-615-3p stem-loop	GTCGTATCCAGTGCAGGGTCCGAGGTATTCGCACTGGATACGACAAGAGG
MTHFD1L forward	TGTGGTGGCTCTGAATGTCT
MTHFD1L reverse	AGGAACTGGAATCGGCTTCT
RPN6 forward	TTCCAGAGTACAGATTGAACACAT
RPN6 reverse	CCAGAGCAGCTTCGTAAGTT
U6 forward	CTCGCTTCGGCAGCACA
U6 reverse	AACGCTTCACGAATTTGCGT
GAPDH forward	GGTGTGAACCATGAGAAGTATGA
GAPDH reverse	GAGTCCTTCCACGATACCAAAG
**The sequences of shRNA against specific targets**	
Name	Oligo Sequence (5’ -3’)
sh-circEYA3-1	TATGATGTTCAGAACATCCAG
sh-circEYA3-2	TGTTCAGAACATCCAGCTGGT
sh-circEYA3-3	TGATGTTCAGAACATCCAGCT
sh-RPN6-1	CCGACGTGGAAAGGAAATTAT
sh-RPN6-2	CTGGTGTCTTTGTACTTTGAT
sh-RPN6-3	GCAAGTCAAAGAGCAGAGCAT
**FISH probe sequence**	
Name	Oligo Sequence (5’ -3’)
circ-MTHFD1L	CACCAGCTGGATGTTCTGAACATCATACAG
miR-615b-3p	AAGAGGGAGACCCAGGCTCGGA
**RNA pull-down probe sequence**	
Name	Oligo Sequence (5’ -3’)
Oligo probe	TAACGACGATCATTTGTACG
circ-MTHFD1L	GGATGTTCTGAACATCATAC
