# Supplementary material for: Circular RNA circ-MTHFD1L induces HR repair to promote gemcitabine resistance via the miR-615-3p/RPN6 axis in pancreatic ductal adenocarcinoma
Source: J Exp Clin Cancer Res. 2022 Apr 23;41:153. doi: 10.1186/s13046-022-02343-z (PMC9034615; doi:10.1186/s13046-022-02343-z)
Supplement: Supplementary file 7 — Additional file 7: Table S2. Correlation of circ-MTHFD1L expression with clinicopathologic features of PDAC patients [file 13046_2022_2343_MOESM7_ESM.pdf]

Table S2. Correlation of circ-MTHFD1L expression with clinicopathologic features of PDAC patients

Characterstics	Total	Circ-MTHFD1L High expression (n=48)	Circ-MTHFD1L Low expression (n=48)	*P* value
Age				0.837
⩾60	53	26	27	
<60	43	22	21	
Sex				0.837
male	55	27	28	
female	41	21	20	
BMI				0.393
<25	62	33	29	
⩾60	34	15	19	
Tumor location				0.682
head	52	27	25	
body/tail	44	21	23	
Differentiation				0.024
well/moderate	53	21	32	
poor	43	27	16	
CA19-9				0.014
⩽37 U/mL	42	15	27	
>37 U/mL	54	33	21	
AJCC stage				0.962
II	19	9	10	
III	38	19	19	
IV	39	20	19	

BMI, body mass index; AJCC, American Joint Committee on Cancer (8th).
