# Supplementary material for: Circular RNA circ-MTHFD1L induces HR repair to promote gemcitabine resistance via the miR-615-3p/RPN6 axis in pancreatic ductal adenocarcinoma
Source: J Exp Clin Cancer Res. 2022 Apr 23;41:153. doi: 10.1186/s13046-022-02343-z (PMC9034615; doi:10.1186/s13046-022-02343-z)
Supplement: Supplementary file 8 — Additional file 8: Table S3. Correlation of PFS with clinicopathologic features of PDAC patients. [file 13046_2022_2343_MOESM8_ESM.pdf]

Table S3. Correlation of PFS with clinicopathologic features of PDAC patients

Median PFS：10.0 months				
**Characterstics**	**Total**	**PFS<10.0 months (n=40)**	**PFS⩾10.0 months (n=56)**	***P* value**
Age				0.199
⩾60	53	21	22	
<60	43	19	34	
Sex				0.383
male	55	25	30	
female	41	15	26	
BMI				0.718
<25	62	25	37	
⩾60	34	15	19	
Tumor location				0.580
head	52	23	29	
body/tail	44	17	27	
Differentiation				0.089
well/moderate	53	18	35	
poor	43	22	21	
CA19-9				0.297
⩽37 U/mL	42	15	27	
>37 U/mL	54	25	29	
AJCC stage				<0.001
II	19	2	17	
III	38	12	26	
IV	39	26	13	
Circ-MTHFD1L				0.004
high expression	48	27	21	
low expression	48	13	35	
RPN6				0.013
high expression	48	26	22	
low expression	48	14	34	

BMI, body mass index; AJCC, American Joint Committee on Cancer (8th).
